# Proton-pumping photoreceptor controls expression of ABC transporter by regulating transcription factor through light

**DOI:** 10.1038/s42003-024-06471-4

**Published:** 2024-06-29

**Authors:** Jin-gon Shim, Kimleng Chuon, Ji‐Hyun Kim, Sang-ji Lee, Myung-chul Song, Shin-Gyu Cho, Chenda Hour, Kwang-Hwan Jung

**Affiliations:** 1 Department of Life Science, Sogang University, Seoul, South Korea; 2 Research Institute for Basic Science, Sogang University, Seoul, Korea; 3 Present Address: Pharmacology Department, Northwestern University Feinberg School of Medicine, Chicago, IL USA

**Keywords:** Membrane proteins, Transcriptional regulatory elements

## Abstract

Light is a significant factor for living organisms with photosystems, like microbial rhodopsin—a retinal protein that functions as an ion pump, channel, and sensory transduction. *Gloeobacter violaceus* PCC7421, has a proton-pumping rhodopsin gene, the *Gloeobacter* rhodopsin (GR). The helix-turn-helix family of transcriptional regulators has various motifs, and they regulate gene expression in the presence of various metal ions. Here, we report that active proton outward pumping rhodopsin interacted with the helix-turn-helix transcription regulator and regulated gene expression. This interaction is confirmed using ITC analysis (*K*_*D*_ of 8 μM) and determined the charged residues required. During in vitro experiments using fluorescent and luciferase reporter systems, ATP-binding cassette (ABC) transporters and the self-regulation of *G. violaceus* transcriptional regulator (GvTcR) are regulated by light, and gene regulation is observed in *G. violaceus* using the real-time polymerase chain reaction. These results expand our understanding of the natural potential and limitations of microbial rhodopsin function.

## Abstract (web summary)

The light-dependent proton-pumping photoreceptor Gloeobacter rhodopsin (GR) binds and controls the role of a HTH transcriptional regulator (GvTcR) in activating expression of ATP-binding cassette (ABC) transporters.

## Introduction

In living organisms, photoreceptors have been classified as photosensors, light-sensing photoconverters, or energy-converting photoreceptors. Various photoreceptors trigger photo movements at various levels—organ translocation and intracellular movements—including photoreceptors with functions in higher species such as plants^1^. Phytochromes, which regulate the subsequent adaptation of plant growth and development, are photoreceptors that absorb red and far-red light (600–750 nm). Cryptochromes and phototropins, which enable absorption and regulation of blue light, UV-A and B (320–500 nm), can be used to monitor almost all facets of light in organism^2^. In addition, photosensory complexes exist such as the microbial rhodopsins and the photosynthetic reaction centers, that function as ion transporters, channels, or energy converters. Antenna pigments (e.g., accessory pigments such as carotenoids and chlorophylls) can bind and form a secondary chromophore. The diversity of photoreceptors has been reported as an essential factor for their function and the differentiation within living organisms^3,4^.

Microbial rhodopsin, a type I rhodopsin, is a photoactive retinal-binding protein that is abundant in the natural environment^5^. Microbial rhodopsins comprise seven transmembrane alpha-helices that absorb light through retinal chromophores and function as ion transporters, ion channels, and photosensing transductors^5^. Microbial rhodopsin is found in numerous marine microorganisms that live in the photic zone of oceans and freshwater^6^. Recently, heliorhodopsin (HeR), characterized by its reverse orientation, has been reported to be widely present in organisms^7^. HeR has been shown to bind to photolyase following light exposure, thereby enhancing DNA repair activity, and this activity is regulated by binding to glutamine synthetase^8,9^. Microbial rhodopsin has evolved various functions. Among them, *Gloeobacter* rhodopsin (GR), which is similar to xanthorhodopsin, exerts a proton-pumping function^10^. *Goeobacter violaceus* PCC7421, cyanobacteria lacking the thylakoid membrane and photosystem, replaces the photosystem with GR. Various carotenoids exist in cells and GR binds to them to form a secondary chromophore and broaden its function^11,12^.

ATP-binding cassette (ABC) transporters are a large superfamily of membrane proteins present in all living organisms (e.g. bacteria, archaea, and eukarya, including humans)^13,14^. ABC transporters comprise four parts: two each of membrane-integrating and ATP-hydrolyzing domains^15^. The typical structure of eukaryotic ABC transporters consists of two conserved domains: a transmembrane domain (TMD) and nucleotide-binding domain (NBD). In contrast, the modules are mostly fused to form a single polypeptide chain and bacterial ABC transporters comprise individual subunits in eukaryotic systems. They are involved in various processes such as signal transduction, protein secretion, drug and antibiotic resistance, antigen presentation, bacterial pathogenesis, sporulation, and nutrient uptake in bacteria^16^. Moreover, they have a multidrug extraction function of toxic substances, which can lead to resistance of cancer cells to drugs used in chemotherapy.

Various mechanisms regulate gene expression, and genes are regulated under specific conditions^17^. Helix-turn-helix (HTH) regulatory proteins regulate gene expression and are classified into two functional types as: activators or repressors. They are distinguished by the positively and negatively regulated transcription of target genes as activators and repressors, respectively^18^. The HTH transcriptional regulator (*bmrR*) of *Bacillus subtilis*, MerR family, is a group of transcriptional activators that regulates *bmr*, the multidrug resistance gene. In contrast, the HTH transcriptional regulator (*zntR*) of *Escherichia coli* regulates the *zntA* transport gene^19^. The HTH transcriptional regulatory proteins, with an abundant motif present in proteins, respond to stress induced by heavy metal toxicity or gene expression regulation without metal ions^20^. The HTH transcriptional regulator binds with metal, fatty acid, tetracycline, and various substances to regulate their genome and to allow the expression of resistance genes to remove, detoxify, or neutralize xenobiotics and ultimately enabling living organisms to survive in harsh environments^21^.

Here, microbial rhodopsin is hypothesized to be involved in gene regulation; therefore, various transcription regulators in *G. violaceus* PCC7421 were investigated. Among the microbial rhodopsins, we found that Heliorhodopsin can function by binding to other proteins, which gave us clues to the binding of transcriptional regulators and microbial rhodopsins^8,9^. We aimed to understand the molecular mechanism of gene regulation driven by non-metal-mediated transcriptional regulators under the influence of photoreceptors.

## Results

### Characterization of GvTcR as HTH-type transcriptional regulators and two promoters that can bind GvTcR

**Fig. 1:**
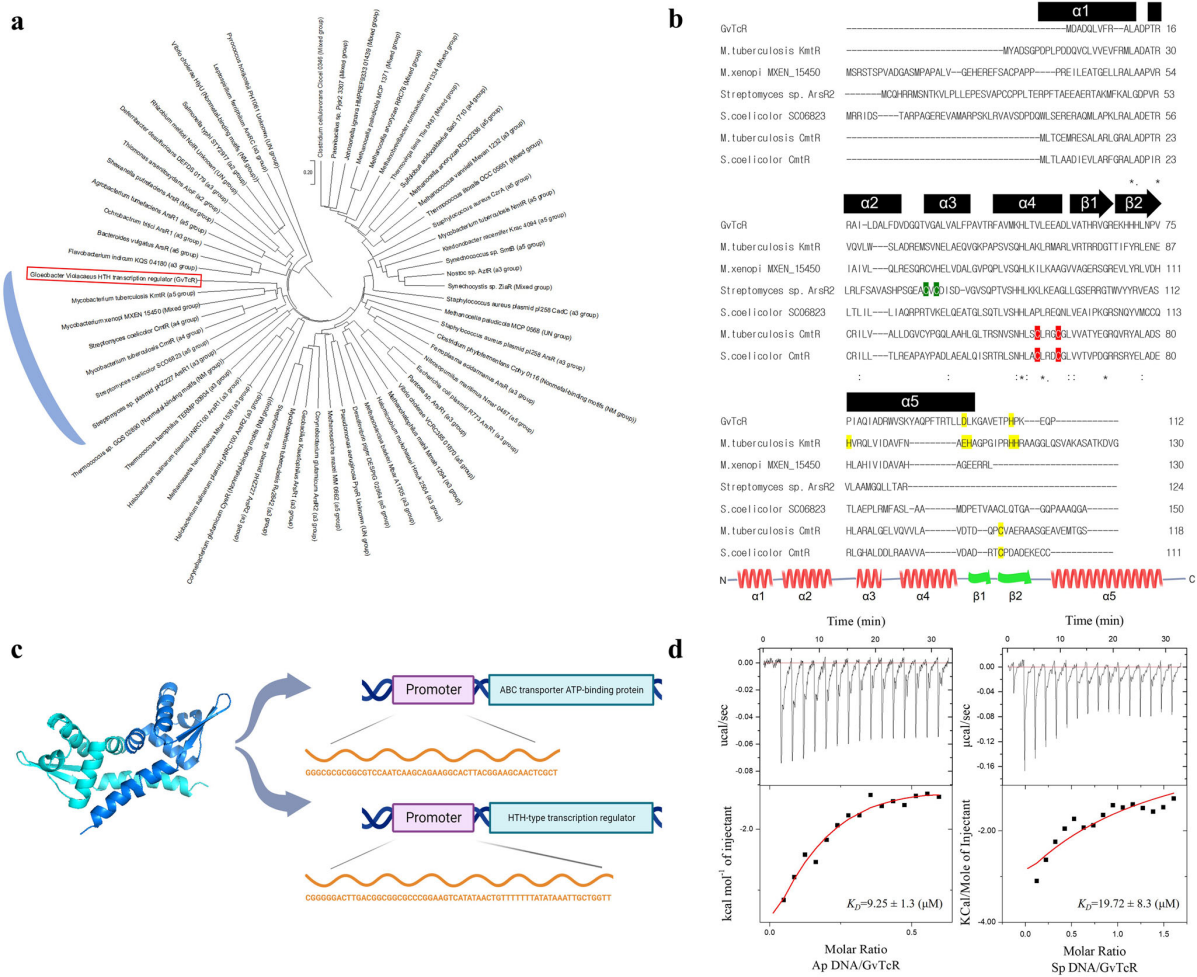
Characterization of GvTcR. **a** The phylogenetic tree of GvTcR with HTH-type transcription regulators. The evolutionary history was inferred using the UPGMA method^34^. The displayed tree is optimal and drawn to scale, with branch lengths in the same units as those of the evolutionary distances used to infer the phylogenetic tree. Poisson correction method was used to compute the evolutionary distances^35^ in units of the number of amino acid substitutions per site. Sixty-one amino acid sequences were analyzed. All ambiguous positions were removed for each sequence pair (pairwise deletion option). A total of 347 positions were found in the final dataset. Evolutionary analyses were conducted using MEGA-X^36^. The blue round box indicates classification as a similar group. The red box indicates GvTcR. **b** Alignment of the GvTcR amino acid sequence with a similar group. Five alpha helix and two beta strands are marked in black boxes. Amino acids that are important for metal bonds are colored. α3 type is indicated in green, α4 type in red, and α5 type in yellow. NCBI reference numbers are as follows: *Corynebacterium glutamicum* CyeR; WP_003855203.1; *Mycobacterium tuberculosis* CmtR, WP_072511063.1; *Streptomyces coelicolor* CmtR, WP_011027409.1; *Mycobacterium tuberculosis* KmtR, WP_049955289.1; *Mycobacterium xenopi* MXEN_15450, WP_099868832.1; *Streptomyces coelicolor* SCO6823, MYU46328.1; *Streptomyces sp*. plasmid pHZ227 ArsR2, ABB70172.1 **c** Analysis of structural simulations based on ArsR-based transcriptional regulators (PDB:3F6O). Expected dimers are marked in different colors. The schematics of the promoter region are displayed. **d** Isothermal titration calorimetry (ITC) analysis shows the binding results for GvTcR with DNA fragments of promoter regions. The top and bottom panels show the raw data and the enthalpy changes. The fitting result is shown in the bottom panel as a continuous line. These experiments were performed at room temperature.

We searched for transcriptional regulators in *G. violaceus* PCC7421 and found that the genetic sequences of HTH-type transcriptional regulators were similar to those in the operon included Heliorhodopsin gene, and we named the candidate as *G. violaceus* transcriptional regulator (GvTcR). We classified GvTcRs as HTH-type transcriptional regulators based on similar genetic sequences with reported HTH-type transcriptional regulators and structure predictions (Fig. 1). To investigate the information of GvTcR (NCBI accession number: WP_011141437.1), various HTH-type transcriptional regulators were compared based on phylogenetic trees (Fig. 1a). GvTcR was classified in a group such as KmtR and CmtR, which are metal-responsive transcription repressors that act by binding to promoter regions, and nickel-, cobalt-, cadmium-, and lead-influenced transcription control was expected.

For the unambiguous classification of HTH-type transcriptional regulators, the structural properties of GvTcR were investigated. GvTcR is structurally composed of five alpha helices and two beta strands and has the same composition as other HTH-type transcription regulators (Supplementary Fig. S1, Fig. 1b)^22^. Analyzing structural simulations based on ArsR-based transcriptional regulators (PDB:3F6O), the presence of the N-terminal arm, a region that forms dimers and plays an important role in DNA binding, was also confirmed on structure and sequence analysis (Fig. 1b, c)^23^. Unlike those classified as a similar sequence to that of KmtR, ITC_200_ analyses confirmed that GvTcR was not bound to cobalt, nickel, or other metals; thus, suggesting that it could be adjusted by factors other than metals (Supplementary Fig. S2). Although presenting a similar sequence to that of KmtR, GvTcR showed no motif at the 5^th^ alpha helix when comparing the major motif sites to which metals bind. In addition, no metal-binding amino acids compared to those in the α3 and α4 groups (Fig. 1b). As the genetic sequence of GvTcR is similar to that of KmtR, we prioritized the ABC transporter ATP-binding protein, a gene regulated by KmtR, as a candidate. We searched for the ABC transporter ATP-binding protein present in *G. violaceus* PCC7421 and shortlisted it by comparing it with genes regulated by KmtR. To identify the genes regulated by GvTcR, ABC transporter ATP-binding protein that was referenced at DNA sequences encoding the regulatory genes of KmtR were compared. Further, promoter candidates containing palindromic sequences were selected for areas before the ABC transporter ATP-binding protein gene (NCBI accession number WP_011141998.1). In addition, self-regulation of the GvTcR gene might be possible, and a total of five candidate DNA sequences were explored through promoter prediction (Supplementary Fig. S3). Five candidate groups were compared using isothermal titration calorimetry (ITC), and as predicted, we confirmed that the ABC transporter ATP-binding protein gene and the promoter region encoding GvTcR bind to GvTcR. The dissociation constant (*K*_*D*_) of the ABC transporter ATP-binding protein coding promoter (Ap) was 9.25 ± 1.3 μM. In contrast, the dissociation constant (*K*_*D*_) of the self-regulated GvTcR coding promoter (Sp) was 19.72 ± 8.3 μM (Supplementary Fig. S3, Fig. 1d). Genetic sequence and structural comparison of GvTcR and thermodynamic analysis of the predicted promoter region showed that GvTcR would function as an HTH-type transcription regulator.

### Interaction between GR and GvTcR by photochemical analysis

**Fig. 2:**
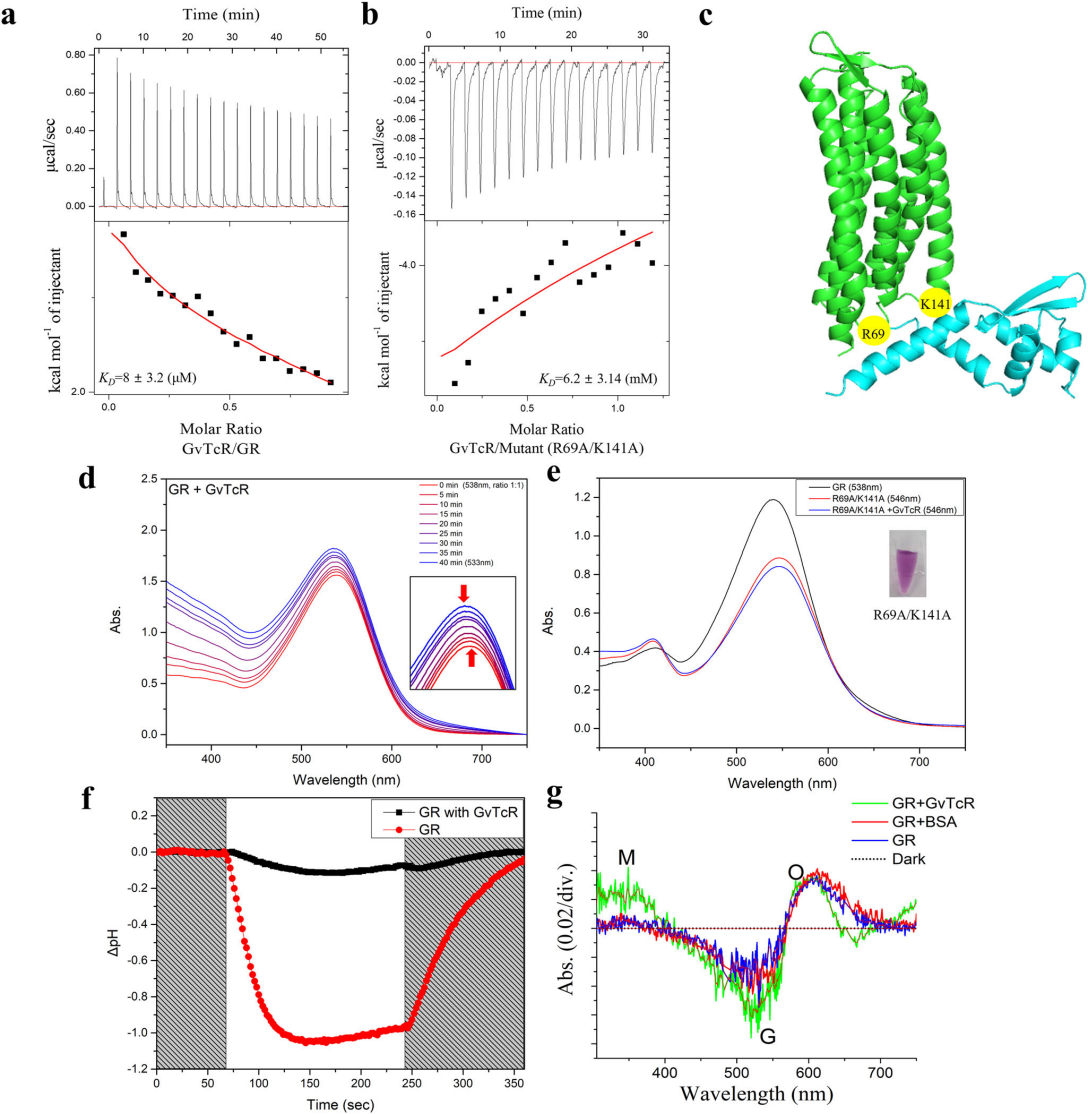
GR interaction with GvTcR and comparison results for mutant isotypes. **a** Isothermal titration calorimetry (ITC) analysis shows the binding results for GR with GvTcR. The top and bottom panels show the raw data and the enthalpy changes. The fitting result is shown in the bottom panel as a continuous line. **b** ITC analysis shows the binding results for R69A/K141A double mutant with GvTcR. These experiments were performed at room temperature. **c** Protein–protein docking simulations (ClusPro 2.0) were performed to predict the binding of GR and GvTcR. Two amino acid sites capable of polar interaction (R69 and K141) are marked with yellow circles as predictions of binding from the intracellular side. **d** The absorption spectra of GR and GvTcR were measured by adding GvTcR (ratio 1:1) to observe the spectral shift for 40 min. **e** The spectral shift of the R69A/K141A double mutant. For the wild type, a blue shift was observed. The mutant GvTcR molar ratio was 1:1, and no shifts were observed. **f** Light-driven proton pumping activities of GR and GR co-expressed with GvTcR. The pumping data purifies GR from each sample after measurement and calculates the same GR expression ratio. The red-dotted line indicates the pumping activity of GR under white light illumination in the presence of 10 mM NaCl. The black-dotted line represents the GR result upon co-expression with GvTcR. **g** Time-based kinetic study of purified GR with and without GvTcR showed light-dependent molecular conformational change with a clear accumulation of red-shifted species at approximately 600 nm representing O intermediates, and the depletion of the green region while the dark-adapted sample was used as a baseline for G intermediates. The green line indicates the result for GR + GvTcR, the red line indicates the result for GR + BSA, and the blue line indicates GR. The dotted line indicates the dark condition.

The function and photochemical properties of GR was hypothesized to be affected when GR interacts with GvTcR and photochemical and photophysical analysis was designed. For comparison, the binding to GvTcR with another membrane protein, proteorhodopsin (PR), was measured by ITC analysis, and the results observed no binding to PR (Supplementary Fig. S4). However, it interacted with GR, with a dissociation constant (*K*_*D*_) of 8 ± 3.2 μM (Fig. 2a). To measure the effectiveness of the combination of the two proteins on the retinal Schiff base, spectroscopic analysis was performed, and the data was observed to be blue, shifting from 538 nm to 533 nm (Fig. 2d). We obtained similar results for the interaction of HeR and the transducer^8^. It influences the chromophore due to the structural change caused by the relatively strong binding of the two proteins with a reasonable *K*_D_^24,25^.

GR is proton outward pumping rhodopsin, and the bond with GvTcR was presumed to influence the pumping ability. To co-expression of protein, a vector that can safely control polycistronic gene expression was prepared by introducing a ribosome-binding site (RBS) into a protein expression vector^26^. We observed that the pumping ability was significantly decreased when GR was co-expressed with GvTcR (Fig. 2f). Further, the purified GR-GvTcR complex from a co-expressed cell was measured in blue-shifted maximum absorption spectra using spectroscopic analysis (Supplementary Fig. S5). Similar to the spectral data, when the two proteins were mixed, a blue shift of 5 nm was observed for the complex (Fig. 2d). To specify protein binding sites, protein-protein docking simulations (ClusPro 2.0) were performed to predict the binding of GR and GvTcR. Following the protein docking simulation, the results of the binding between the two proteins were obtained, and mutation sites that played an important role in the binding of the two proteins were explored^27^ (Fig. 2c). As three amino acids (R69, K141, and R202) of GR can be involved in polar interactions, the R69, K141, and R202 positions were selected as mutation candidates (Supplementary Fig. S6, S7 left lane). The three candidates were substituted with alanine. Although the R202A mutant showed no difference in *K*_D_ compared to the wild-type, the R69A mutant exhibited a more than 10-fold increase in *K*_D_ compared to that of the wild-type (Supplementary Fig. S6). The K141A mutant also affected protein binding and the R69A/K141A double mutant was analyzed using ITC (*K*_*D*_ = 6.2 ± 3.15 mM) (Fig. 2b). The *K*_D_ of the R69A/K141A double mutant increased by approximately 10^3^. We confirmed that the shift did not occur differently from the wild type by comparing the spectral shift results for the combination of the R69A/K141A double mutant and GvTcR (Fig. 2e). This suggests that the positions of R69 and K141 are crucial for protein binding. In addition, changes in protein bands were expected by gel electrophoresis for protein-protein interactions. The binding of GR to GvTcR with and without His tag was compared. The GRs were found to be shifted upward compared to the single GR when the cell lysate containing GvTcR without His tag flowed through. This is a binding-dependent gel shift and considering that the GR R69A/K141A mutant binds weakly, we confirmed the results for cell lysates expressing His tagged GvTcR. In the GR R69A/K141A mutant, the band of GvTcR was measured but no shift occurred. Also, PR did not bind to GvTcR and no band of GvTcR was shown. The binding of GR to GvTcR was measured by pull-down assay, and the mutant showed weak binding (Supplementary Fig. S7 right lane).

The resulting blue shift of the GR-GvTcR complex can affect the retinal Schiff base through protein binding, which can affect the intrinsic photocycle of GR. A time-based kinetic study of purified GR with or without GvTcR showed light-dependent molecular conformational changes with an accumulation of red-shifted species at approximately 600 nm, representing O intermediates (Fig. 2g). The green light region was depleted, while the dark-adapted sample was used as the baseline for the G intermediates. In particular, we observed a significantly higher amplitude signal of the blue absorption region, which is estimated to represent the M-like intermediate in the presence of GvTcR compared to those in the presence of GR or GR + BSA (bovine serum albumin) alone (as a control group that does not bind proteins to GR). Absorption changes were continuously assessed under light and darkness at 600, 535, and 355 nm to further investigate the kinetic changes. Interestingly, for GR, GR + BSA, and GR-GvTcR complex, the excitation and relaxation of the 600 nm absorption band were similar in all cases, while for GR and GR + BSA, the corresponding rapid recovery was observed at 535 nm. However, for the GR-GvTcR complex, the absorption recovery was much slower at 535 nm, and the blue band was gradually excited and relaxed at 355 nm, which might represent a deprotonation-like state. A light-dependent differential spectrum scan with higher absorption amplitudes was observed in the blue region (Supplementary Fig. S8). These results suggest that active proton-pumping rhodopsin can bind to transcription regulators and that photochemical properties change through interactions.

### GvTcR- and GR-mediated gene regulation

**Fig. 3:**
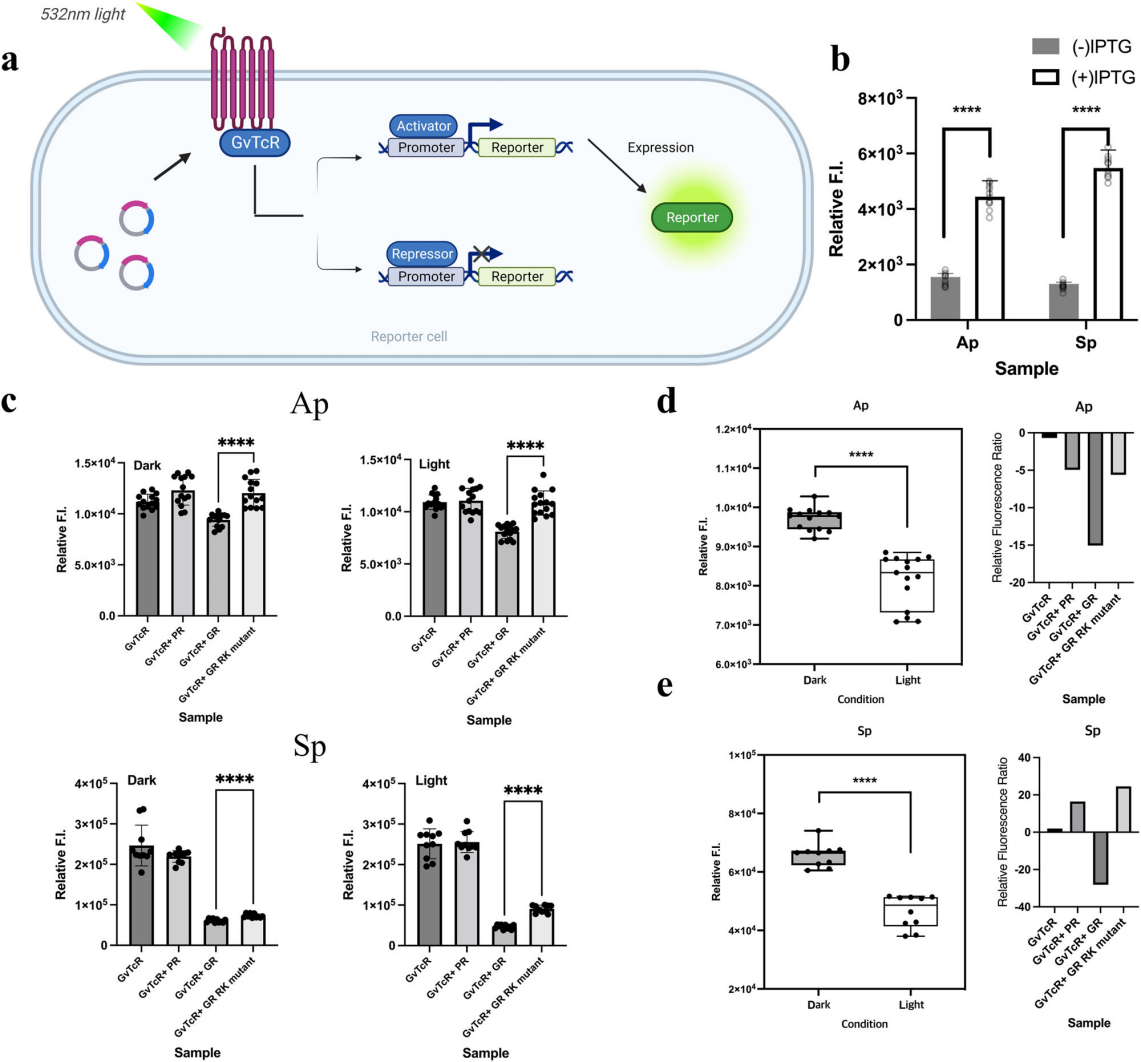
In vivo experiments using the fluorescent reporter system. **a** Schematic representation of the fluorescent reporter system. GR and GvTcR co-express proteins using cloned vectors. Each promoter (Ap, Sp) is cloned before the reporter protein. Green fluorescent protein (GFP) is expressed as a function of the presence or absence of light. It is a system in which the fluorescence level increases for an activator and decreases for an inhibitor. **b** Reporter system results are meant to verify the functionality of GvTcR. A bar graph indicates the fluorescent protein changes depending on isopropyl β-D-1-thiogalactopyranoside-induced protein expression. The results for the dark condition are represented by a dark gray bar, and the results for the light condition are represented by a white bar. *P*-values were assessed using a Student’s *t* test (****P < 0.001; *n* = 15 biologically independent samples) **c** Bar graph comparing fluorescence levels due to GR and GvTcR co-expression in two promoters (Ap and Sp). The upper lane represents the Ap results, and the lower lane represents the Sp results. The graph is marked according to dark and light conditions. When only GvTcR is present, dark gray is used as a differentiator; GvTcR + PR is marked in bright gray; GvTcR + GR is marked in grey; GvTcR + R69A/K141A double mutant is marked in white (****P < 0.001; *n* = 15, 10 biologically independent samples). **d**, **e** Comparison plot box chart for the dark and light conditions at the Ap and Sp promoter from (**c**) (left lane). Dark grey represents the dark condition result while white represents the light condition results. The right lane showed the differences in relative fluorescence ratio at the dark and light conditions for the four sample results. Samples are marked separately on the *x*-axis. *n* = 15 biologically independent samples. All samples with multiple n’s are labeled with an error bar. The standard error is calculated by dividing the standard deviation by the square root of number of measurements.

After analyzing the binding results of GR and GvTcR, we hypothesized that GR could regulate the GvTcR gene expression. Hence, we subsequently evaluated whether GvTcR can regulate genes by binding to DNA and transducers. Alternatively, fluorescent reporter gene analysis designed that GvTcR can regulate the ABC transporter ATP-binding protein gene and the GvTcR gene^28,29^. If each promoter (Ap, Sp) was operated through GvTcR, the reporter gene, green fluorescent protein (GFP), was expressed (Fig. 3a). GvTcR bound to each promoter and increased the expression of GFP, indicating that GvTcR serves as an activator (Fig. 3b). For the comparative experiment, when the non-transformed cells, the only promoter-transformed cells, and the only GvTcR-transformed cells were compared, we observed that promoters were operating weakly, showing a low fluorescence level when only the promoter was introduced. Furthermore, the activation was increased by GvTcR (Supplementary Fig. S9). Candidate promoters (1 P, 3 P) that do not bind GvTcR showed relatively low fluorescence levels. These results suggest that Ap and Sp can act as promoter regions and be regulated by GvTcR. PR was used to analyze whether GvTcR, could bind to GR as an activator (Supplementary Fig. S10). For PR, no differences in the GFP expressed were observed following the addition of GvTcR (with or without the retinal and light source).

Differences in light-induced reporter protein expression were observed when GR was present in the promoter (Ap) of the ABC transporter ATP-binding protein gene and that of the GvTcR gene (Sp) (Fig. 3c). Interestingly, the GFP expression level decreased under dark conditions, suggesting that the gene regulatory function of GvTcR can be affected only by binding to GR. In the case of Ap, it decreased by approximately 20% compared to that observed in the control group (only GvTcR, GvTcR with PR) under dark conditions. In comparison, it decreased by approximately 35% under light conditions (Fig. 3c upper lane). A difference of approximately 15% was observed between the dark and light conditions (Fig. 3d). Alternatively, similar results were obtained from the R69A/K141A double mutant, as for the control group. This indicates that the function of GvTcR could be controlled by light following the interaction with GR. When GR was present with the Sp promoter, the GFP expression rate was reduced by approximately four times under dark conditions. While in light conditions, it decreased by approximately 25% compared to dark conditions (Fig. 3c lower lane, 3e). Nevertheless, the R69A/K141A double mutant was associated with different results from the wild type when GR was present in Ap. The GFP expression rate of the R69A/K141A double mutant also decreased, such as of the wild-type; however, the GFP expression rate was higher than that of the wild-type under light conditions. This might be due to GR binding as observed from the slight differences in *K*_*D*_ values of the R69A/K141A double mutant when compared to those of the wild type. GvTcR has a stronger effect on Sp compared to Ap, suggesting that differences in regulatory levels can be seen in regulating genes. Figure 3d, e (bar graphs at the right) observed slight light effects with the inactive proteins (PR and GR mutant). However, inactive proteins (PR and GR mutant) showed weak binding, which is considered residual activity through this weakened binding.

**Fig. 4:**
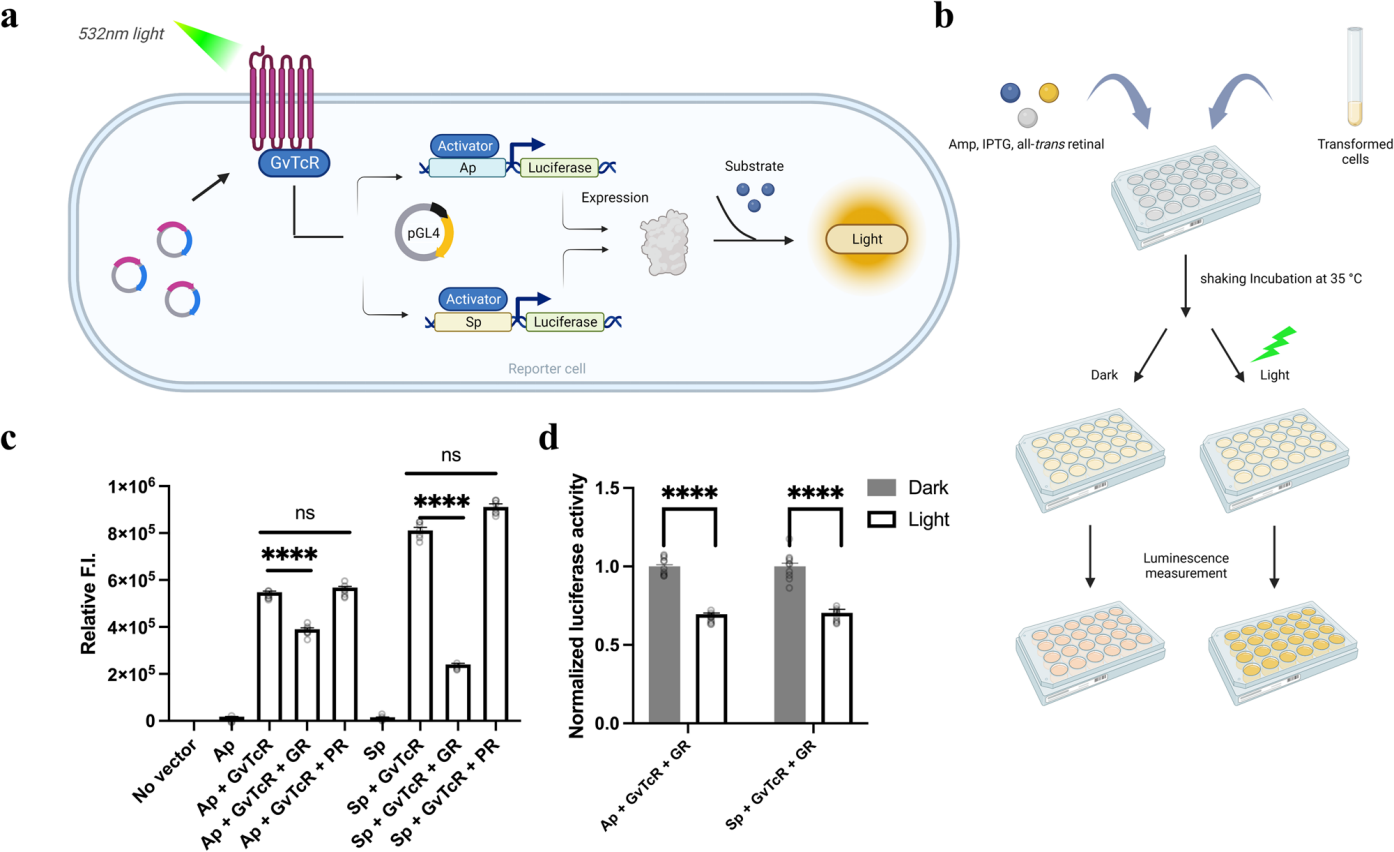
In vivo experiments using the luciferase reporter system. **a** Schematic representations for the luciferase reporter system. GR and GvTcR express proteins using cloned vectors. Each promoter (Ap, Sp) is cloned in front of the reporter protein. Luciferase is expressed according to the presence or absence of light. It is a system in which the luminescence level increases for an activator. **b** Schematic representation of a 24-well plate experimental course of the luciferase reporter assay. Ampicillin, IPTG, and all-trans-retinal inclusion are indicated in circles. **c** The relative luminescence intensity according to each condition is indicated in a bar graph. In the presence of GvTcR alone, high luminescence levels were observed; however, in the case of samples containing PR, non-significant changes in luminescence were observed when compared to those measured in the presence of GvTcR alone. *P*-values were assessed using the Student’s *t* test; ****P < 0.001. *n* = 8 biologically independent samples. **d** Graph comparing normalized luciferase activity in the dark and light conditions when GR exists according to each promoter condition. The dark condition is illustrated in the dark grey box and the light condition is indicated in the white box (****P < 0.001). *n* = 8 biologically independent samples. All samples with multiple n’s are labeled with an error bar. The standard error is calculated by dividing the standard deviation by the square root of number of measurements.

A luciferase reporter system confirmed that GvTcR could bind to GR and regulate genes through light (Fig. 4a). We confirmed that the reporter protein was expressed by expressing GvTcR with Ap and Sp. such as the fluorescent reporter system, weak signals appeared only when promoters were present. When GvTcR was co-expressed, high luminescence levels were obtained regardless of the presence or absence of light (Supplementary Fig. S11). To further analyze this aspect, transformed reporter cells were cultured, and induced protein expression in a 24-well plate, following the presence or absence of green light (532 nm) (Fig. 4b). Similar to the fluorescent reporter system, the luminescence level decreased meaningfully when GR was present in the two promoter regions (Fig. 4c). In the presence of GR and GvTcR, the luminescence level decreased by approximately 20% for Ap, while for Sp, it decreased by more than 65%. In the presence of PR with GvTcR, luminescence levels were similar to those measured for GvTcR alone. Hence, when GR is present in the Ap or Sp regions, a decrease of approximately 35% can be observed under light conditions (Fig. 4d). Similar to the fluorescent reporter system, in the luciferase reporter system, GR binds and affects to GvTcR even in the dark. However, a further decrease of approximately 35% under light conditions suggests that GR modulates GvTcR and exerts stronger control by light. These results suggest that the gene regulatory function of GvTcR is affected by GR in the presence or absence of light. These results suggest that the gene regulatory function of GvTcR is affected by the presence or absence of light; however, it is relatively more affected by the binding of the GR itself.

### Transcriptional Regulation of GR and ABC transporters by real-time PCR with *Gloeobacter violaceus* PCC7421

**Fig. 5:**
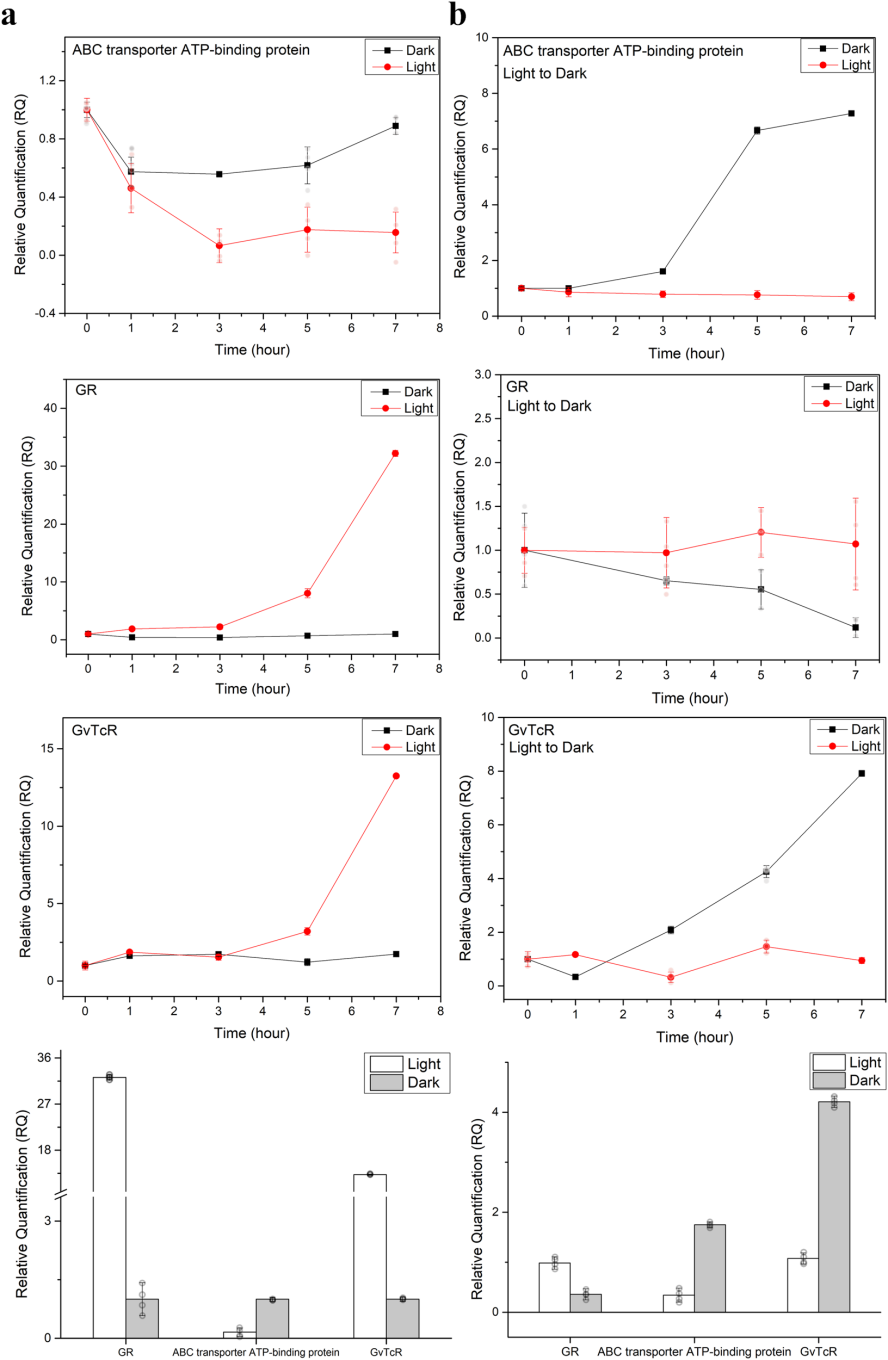
Real-time PCR of *G. violaceus* PCC7421. Line plot graph comparing relative quantification values for each condition. **a** After three days of adaptation to dark conditions, it was measured by irradiating light (red line plot graph), and samples maintaining dark conditions were compared to controls (black line plot graph). GR and GvTcR transcriptions were confirmed using PCR and the prepared primer sets. The bottom panel represents a bar graph comparing dark and light conditions at 7 h. *n* = 4 biologically independent samples. **b** After three days of adaptation to light conditions, it was measured in dark conditions (blackline plot graph). It was compared through a red line plot graph maintaining light conditions. The bottom panel represents a bar graph comparing dark and light conditions at 7 h. *n* = 4 biologically independent samples. All samples with multiple n’s are labeled with an error bar. The standard error is calculated by dividing the standard deviation by the square root of number of measurements.

The interaction of GR and GvTcR changes the photochemical properties and affects the proton pumping activity of GR. In addition, GvTcR was found to act as an activator in the promoter region of the two genes through the reporter system and the activator is suppressed in the presence or absence of light through GR. Unlike experiments in *E. coli*, *G. violaceus* PCC7421 has the possible effect of the carotenoid antenna pigment of GR. It has been reported that GR increases the light-harvesting function by combining with carotenoids such as salinixanthin and canthaxanthin (CAN), which increases the efficiency of the energy absorbed. We assumed that the light-harvesting function would not significantly affect the binding of GR and GvTcR. It was assumed that changes in mRNA levels would be largely influenced by the combination of GR and GvTcR rather than by the carotenoids of *G. violaceus* PCC7421. Real-time PCR was designed to indirectly examine the effect of the presence or absence of light on the regulation through GR and GvTcR in *G. violaceus* PCC7421. RT-PCR was used to study whether GR can control gene expression, which was assumed to be controlled through a variety of complex processes. Time-course measurements were performed while illuminating the organism in the dark. Time-course measurements were performed after changing from light conditions to dark conditions. Primers for each target gene, GR, ABC transporter ATP-binding protein, and GvTcR genes, were tested by concentration to form a single band (Supplementary Fig. S12). We observed a change when transitioning from dark to light conditions, and the mRNA level of the ABC transporter ATP-binding protein decreased to levels that could rarely be measured after 3 h (Fig. 5a). In the case of GR, the mRNA level began to increase after light irradiation and increased up to 45 times until 7 h. The mRNA level of the GvTcR gene showed an increase at 5 h and was measured up to 14 times. The mRNA level of the GvTcR gene was controlled by the increase in the mRNA level of GR, which was hardly measured during the early stages, and the mRNA level of the ABC transporter ATP-binding protein showed a rapid decrease. Although the transcriptional regulation of GvTcR was suppressed during the early stages of expression, it increased during later stages, thus suggesting that the GR regulation inhibits GvTcR transcription during early stages. In addition, the expression rate of GR transcription increased with light irradiation, suggesting that it operates as a photosystem.

The transcription level of the ABC transporter ATP-binding protein gene increased up to 8 times when transitioning from the light to the dark condition (Fig. 5b). In the case of GvTcR, the mRNA level increased faster under dark conditions than under light conditions, and when dark conditions were maintained, no changes were observed over time. The transcription of GR slowly decreased as the light disappeared and we were rarely able to measure it after approximately 7 h. Consequently, the transcriptional level of GR was increased by light, and the transcriptional levels of GvTcR and ABC transporter ATP-binding protein genes were repressed during the initial phase of GR transcriptional increase compared to the results measured after 5 h. However, the transcription level of GvTcR and the ABC transporter ATP-binding protein gene was observed to increase under dark conditions. In contrast, the transcription level of GR decreased because the role of the photosystem was not expected in dark conditions. Comparing the transcript levels of GR and GvTcR after 5 h, the transcript levels of GR were measured to be approximately 3 to 4 times higher than those of GvTcR. It suggests that as the transcriptional level of GR comes to exceed that of GvTcR, the transcriptional level of GR increases for light-induced energy production, which simultaneously causes the energy used by GvTcR and the ABC transporter ATP-binding proteins to decrease to capture more of the through light-energy produced ATP for driving metabolic processes.

## Discussion

Globobacter rhodopsin (GR), identified as a light-induced proton pump, has also been shown to have an affinity for the transcriptional regulator GvTcR. As a result, the proton pump function of GR in the complex is strongly inhibited. Evidence was presented that this complex can also form in the native organism *G. violaceus* PCC7421 and that it can reciprocally regulate the expression of its components and ABC transporters, one of the targets of GvTcR, in a light-dependent manner. This study started from the idea that HTH-type transcriptional regulators can bind to GR. In *G. violaceus* PCC7421, GR acts as a photosystem for energy production and shows functional evolution in combination with various carotenoids^11,30^. GvTcR, capable of binding to GR, was obtained by exploring HTH-type transcription regulators, such as genes present in living organisms. Unlike KmtR and CmtR, which have similar sequences with GvTcR, GvTcR does not bind to metals and is assumed to be regulated by GR. This might be underlined by an evolutionary flow in which HTH-type transcription regulators express a defense mechanism against low-concentration harmful metals in the natural environment through light. The concentration of harmful metals is remarkably low, and the function of removal and avoidance can be achieved by effectively controlling them through light. The ABC transporter ATP binding protein and GvTcR genes that can be controlled with GvTcR were identified and controlled by interaction with GR (Figs. 3, 4). Through structural and binding simulations, we identified two amino acids, R69 and K141, that are involved in the binding of GvTcR to GR and confirmed that these are critical sites. In addition, the GR R69A/K141A mutant was measured to have a 1000-fold weaker *K*_*D*_ than GvTcR and a pull-down assay was performed to compare it to the gel electrophoresis results (Figure S7). Cell lysates expressing GvTcR without His tag were run on GR, which showed an up-shifted band. For the GR R69A/K141A mutant, we flowed His-tagged GvTcR-expressing cell lysate to account for weaker binding and saw a band of GvTcR but no up-shifted band. In the case of PR, the band of GvTcR and the shifted band were not visible, suggesting that GvTcR does not bind to PR.

**Fig. 6:**
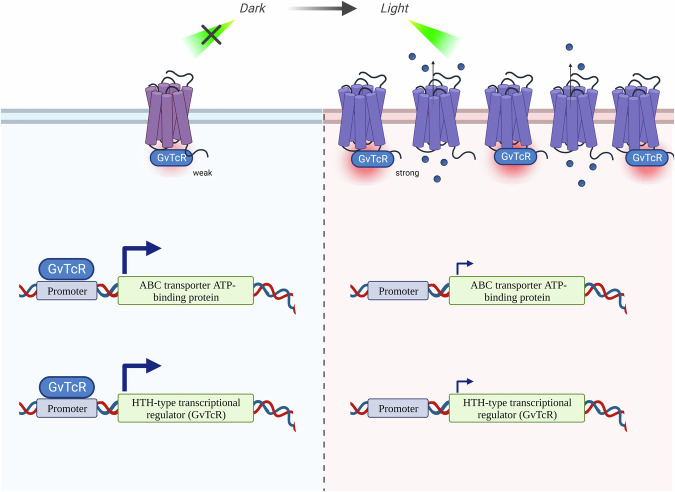
Schematic representation of the mechanism through which GR regulates GvTcR through light. The transcription of GR is increased in the presence of light, thereby suppressing GvTcR expression. The target genes of GvTcR, ABC transporter ATP binding protein, and self-regulation are suppressed and a sufficient number of GRs are present to maintain energy production function. Under dark conditions, the transcription of GR does not increase; however, the existing GRs regulate the gene expression of GvTcR.

We observed that the binding of GR with GvTcR reduced pumping activity (Fig. 2f), thereby replacing its function as a sensor through the photocycle measurement. The binding of GR to GvTcR results in the appearance of a slowed M-like intermediate. This suggests that a conformational change in the M-like intermediate limits the rate of SB deprotonation, and this conformational transition is the inward bending of the transmembrane helix C toward helix G as the water molecules rearrange along the proton potential channel^31^. GR binding to GvTcR involves R69 and K141, and these amino acids are present in intracellular loops 1 and 2 centered on helix C. This suggests that the movement of helix C is disrupted by bound GvTcR. For this reason, we propose that it affects the M-like intermediate and slows it down. In addition, R69 and K141 are critical for GvTcR binding, which in turn affects the uptake of proton. This would lead to the binding of GvTcR to membrane proteins, inhibiting the opportunity for gene regulation. GvTcR influences the proton transfer to an acceptor from the retinal Schiff base, hindering the proton pumping activity, and introducing a conformation change that extends the first conformation change when excited energy is applied to the sensor. Whether this conversion as a sensor can be regulated in *G. violaceus* PCC7421 was investigated using RT-PCR. Experiments in *G. violaceus* PCC7421 may affect carotenoid antenna pigments on GR. GR interactions with carotenoids such as salinixanthin and canthaxanthin (CAN) form a secondary chromophore. We presumed that increasing light absorption efficiency by forming a secondary chromophore would not significantly affect the binding of GR and GvTcR. In addition, there are limitations in controlling the expression of GR and GvTcR through carotenoid binding in the *E. coli* expression system; hence, further research is needed. In dark conditions, the mRNA level of GR, which serves as a photosystem, was increased with light exposure. It suggests cells respond by increasing GR transcription for energy production in light. The mRNA level of GR tended to increase by light irradiation; however, the mRNA level of the ABC transporter ATP-binding protein decreased. In the case of the light adaptation state to dark condition. In contrast, the mRNA level of the ABC transporter ATP-binding protein increased, and the transcription level of GR decreased. It suggests that GR regulates the mRNA level of ABC transporters through interaction with GvTcR to generate ATP through light and reduce the consumption of unnecessary ATP for biological reactions. The ABC transporter exports lipids, sterols, drugs, and a large variety of primary and secondary metabolites using ATP. The ABC transporter ATP-binding protein regulates the transcription level for energy production and accumulation, because its function is additive in the biological response for cell survival^14^. While light is being irradiated, the GR aids energy production and adjusts the accumulated energy efficiently to distribute it to various metabolic processes. GvTcR, which continuously increases the amount of self-regulation, is unnecessary energy consumption to cells. Therefore, the mechanisms regulated by GR can be seen as an evolutionary step to implement a GR-controlled mechanism for situations where metal ions cannot be combined. Following the light-induced increasing the mRNA level of GR, the mRNA level of GvTcR is suppressed and might be controlled to prevent unnecessary energy consumption. It is understood that sufficient energy accumulation occurs after light energy has been converted into chemical energy, after which the mRNA levels of GvTcR increase for use in various biological processes. It was also observed in the light adaptation state to dark conditions. In the case of the turned-off the light and the mRNA level was tracked, the mRNA level of GvTcR increased more rapidly than when initially inhibited by the expressed GR (Fig. 5). Interestingly, as shown in Fig. 5a, the mRNA level of GR and GvTcR increased meaningfully starting at 5 h. These results are considered the possibility that if the mRNA level is translated into protein expression, the expression of GR, which is responsible for converting light energy into ATP, may be increased under light conditions. In contrast, the transcription of the ATP-consuming ABC transporter ATP-binding protein may be downregulated. This suggests that GR also regulates the expression of GvTcR to lower the expression of ABC transporter ATP-binding proteins that use ATP for energy in organism. The light-responsive system increases the expression rate of GR, which functions as a proton transporter, and the amount of intracellular ATP increases, and the freed GvTcR increases the expression of GvTcR through self-regulation. The relative quantification of GR was measured to be approximately 4-fold higher than that of GvTcR. This is consistent with a steady increase in GvTcR expression over time, suggesting such a model (Fig. 6).

Based on the model, The transcription of GvTcR is increased for use in various biological processes after converting light energy into chemical energy. Light-dependent transcriptional level regulation of proteins in living organisms provides clues to the circadian rhythm. To supply energy using light, the expression of photoreceptors is increased, which produces ATP and simultaneously suppresses the transcription of other proteins to prevent indiscriminate energy consumption. When a sufficient amount of energy is produced under light, it regulates the transcription of photoreceptors to control excessive energy production for use in various biological processes. In the absence of light, it is suggested to have formed an evolutionary circadian clock that can live in a harsh natural environment by using the produced energy for various biological processes. Interestingly, it is the effect of the presence or absence of light, as well as the presence or absence of GR itself. These results suggest that the gene regulatory function of GvTcR is affected by the presence of light yet is relatively more dependent on the binding of the GR itself. Further studies will be needed to characterize the structural features of GR and GvTcR in light and dark conditions.

In this study, we provided insights into GvTcR functionality, which is moderated by light via GR. GR plays an important role in ATP production as a photosystem and regulates transcriptional levels for energy accumulation. In the dark, the existing expressed GR binds with GvTcR and regulates the transcriptional level of the ABC transporter ATP-binding protein and GvTcR (Fig. 6). In the presence of light, the transcription level of GR is increased in the cell; thus, inhibiting the transcription of GvTcR and reducing the ABC transporter ATP-binding protein. As the transcription level of GR increases, energy is produced due to the increase in proton pumping activity during the inhibition of GvTcR through GR. It is considered to be the process of producing and accumulating energy in the presence of light while efficiently avoiding unnecessary energy consumption. GvTcR regulates the increase in transcription level of the ABC transporter ATP-binding protein and GvTcR for various bioreactions when sufficient energy is accumulated. It is suggested as part of a cyclic biological reaction process according to the presence or absence of light. From these results, the circadian clock process by which GR transcription is increased by light is challenging to explain. It is presumed to be modulated through certain receptors by light, and GR transcription is expected to occur through various processes. In addition, the possibility that the function of GvTcR affects the regulation of other genes and the two genes of this report should also be investigated. In-depth research on the biological meaning of regulating the finally regulated ABC-transporter ATP binding protein is needed. We have continuously studied and reported the function and role of GR in binding with carotenoids to form secondary chromophores. Studies on GRs regulating the transcriptional control of GvTcR suggest that the function of carotenoid-mediated secondary chromophores in live cells may provide a new direction for regulation. The regulation of GvTcR by carotenoid binding of GR requires further study.

## Materials and methods

### Cloning of GR and GvTcR

*Gloeobacter violaceus* PCC 7421 rhodopsin (GR; accession no. NP_923144) and GvTcR (NCBI accession number: WP_011141437.1) were obtained through genomic DNA PCR using specific primers. Forward and reverse primers containing a hexahistidine tag at the C-terminus for GR were 5′- TACATATGTTGATGACCGTAT-3′ and 5′- GGGCGGCCGCTCAGTGATGATGGTGGTGATGGGAGAT-3′, respectively. Forward and reverse primers containing a hexahistidine tag at the C-terminus for GvTcR were 5′- ATCATATGGATGCGGACCA-3′ and 5′-TGCGGCCGCTTAGTGATGATGGTGGTGATGTGGCTGCTC-3′, respectively. The PCR fragments were introduced at the *Nde*I and *Not*I restriction enzyme sites for introduction into the pKA001 vector^32^.

### GR and GvTcR co-expression plasmid construction

The GR and GvTcR genes with C-terminal hexahistidine tags were cloned into the pKA001 vector. GR has *BamHI* and *XmaI*, while GvTcR has *NdeI* and *NotI* restriction enzyme sites. Both genes were located under the lacUV5 promoter, and IPTG induced their expression. GR and GvTcR were sequentially arranged in the vector sequence. An additional ribosomal binding site (RBS) was generated between the GR stop codon and GvTcR start codon to improve GvTcR expression. Each GR and GvTcR fragment was amplified using PCR and the 5′-CGGATCCATGTTGATGAC-3′ (GR forward primer; BamHI), 5′- ATATCTCCTTCTTAAAGTTAAACAAACCCGGGTCAGTGATGATGGTGGTGATGGGAGATAAGAC-3′ (GR reverse primer; 6xHis + *XmaI* + RBS), 5′- GGTTTGTTTAACTTTAAGAAGGAGATATCAtATGgatgcggacc-3′ (GvTcR forward primer; RBS + *NdeI*), and 5′-GTGCGGCCgcttaGTGATGATGGTGGTGATGtggctgctcc-3′ (GvTcR reverse primer; 6xHis + *NotI*) primers. After performing an overlap-extended PCR with the two generated gene fragments to obtain fragments linked to GR and GvTcR, pKA001 vector cloning was conducted using *BamHI* and *NotI* restriction enzymes and T4 ligase.

### Sequence analysis and phylogenetic tree

The similarity of the amino acid sequence of GvTcR to that stored in the NCBI database was determined using BLAST-P. Multiple sequence alignments were constructed using MUSCLE^33^. The 2D structure of GvTcR was predicted using Jpred4^22^. The operons for several genomes of each bacterium containing photolyases were investigated using the NCBI GenBank database. Evolutionary history was inferred using the UPGMA method^34^. An optimal tree is shown. The tree was drawn to scale, with branch lengths in the same units as those of the evolutionary distances used to infer the phylogenetic tree. The evolutionary distances were computed using the Poisson correction method^35^. Evolutionary analyses were conducted using the MEGA-X software^36^.

### Protein expression in *E. coli*

Further, pKA001-GR and pKA001-GvTcR were transformed into *Escherichia coli* UT5600, and single colonies were selected and grown in Luria-Bertani (LB) broth containing ampicillin (50 µg/mL) at 35 °C and 200 rpm overnight. After overnight culture, 1% of the total volume was transferred to a new broth, grown to an optical density (OD) of 0.5 and measured at 600 nm. Protein expression was induced by adding 1 mM isopropyl β-D-1-thiogalactopyranoside (IPTG; Duchefa Biochemie, Netherlands) and 7 µM all-*trans*-retinal (Sigma Aldrich, St. Louis, MO, USA) for 4 h at 35 °C. GvTcR expression was induced using IPTG alone. Cells were harvested by centrifugation for 15 min at 5,000 rpm and 4 °C.

### Purification of rhodopsins

Harvested cells expressed with membrane protein were lysed via sonication with sonication buffer (150 mM NaCl, 50 mM Tris-HCl, pH 7.0) and ultracentrifuged for 1 h at 35,000 rpm and 4 °C (Beckman, Brea, CA, USA) at the Advanced Bio-Interface Core Research Facility, and pellets were resuspended in 1% n-dodecyl-β-d-maltopyranoside (DDM; Anatrace, Maumee, OH, USA) dissolved in sonication buffer (150 mM NaCl, 50 mM Tris-HCl, pH 7.0) overnight at 4 °C for solubilization. After solubilization, centrifugation was performed for 15 min at 20,000 rpm and Ni^2+^ NTA agarose (Qiagen, Hilden, Germany) was added to the supernatant. The mixture was shaken gently for 4 h at 4 °C. The expressed rhodopsins were separated using affinity chromatography using wash buffer (25 mM imidazole, 0.02% DDM, 150 mM NaCl, 50 mM Tris-HCl, pH 7.0) and elution buffer (250 mM imidazole, 0.02% DDM, 150 mM NaCl, 50 mM Tris-HCl, pH 7.0). Purified rhodopsin was concentrated using an Amicon Ultra-4 10 K centrifugal filter tube (Millipore, Burlington, MA, USA). For GvTcR, cells were disrupted through sonication. After removing the cell debris through centrifugation (4000 rpm at 4 °C), the cell lysate was subjected to Ni^2+^-NTA agarose. GvTcR was eluted with sonication buffer containing 250 mM imidazole and subsequently concentrated in an Amicon Ultra-4 10 K centrifugal filter tube.

### Absorption spectroscopy

Absorption spectra were measured using a UV-visible spectrophotometer (UV-2550; Shimadzu, Japan) and their values were analyzed using Origin 9.0.

### Light-driven proton transport assay

The expressed cells were collected by centrifugation at 5000 × *g* for 15 min, suspended in pumping solution (10 mM NaCl, 10 mM MgSO_4_, and 10 µM CaCl_2_), and centrifuged again at 4000 × *g* for 15 min (Eppendorf centrifuge 5810 R, Germany). The expressed whole cells were washed with a pumping solution for each measurement^37^. The cells were illuminated by a shortwave cutoff filter (>440 nm, Sigma Koki SCF-50S-44Y, Japan). The pH variation was monitored (Horiba pH meter F-71A, Japan), and data were recorded using the LAQUA Software Ver. 1.47 (Japan). Three measurements were performed for each sample to compare the degree of proton pumping and the average value was calculated.

### Binding affinity was determined using isothermal titration calorimetry (ITC)

For ITC analysis^12,38^, wild-type and mutant GvTcR were completely replaced with sonication buffer containing 0.02% DDM using Amicon Ultra-4 10,000 MWCO centrifugal filter units. ITC analysis was performed using a MicroCal ITC200 instrument (Malvern Panalytical, UK). Data analysis was performed using the Origin-ITC software. Gene sequences in promoter regions that can bind GvTcR. Each sequence was inserted using the 5′-GGGCGCGCGGCGTCCAATCAAGCAGAAGGCACTTACGGAAGCAACTCGCT-3′, and 5′-CGGGGGACTTGACGGCGGCGCCCGGAAGTCATATAACTGTTTTTTTATATAAATTGCTGGTT-3′ primers.

### The spectral shift upon GR and mutant binding to GvTcR

Purified GR and GvTcR were mixed in a 1:1 molar ratio and time-dependent absorption spectra were measured using a UV-2450 spectrometer. Purified GR mutant and GvTcR were mixed in a 1:1 molar ratio. Based on the complex formation ratio by isothermal titration calorimetry, a GR-GvTcR complex was formed via 1:1 binding. It was estimated that under these conditions about 60% of the total GR present was bound in a GR-GvTcR complex.

### Time base kinetic experiment and ultra-kinetic

Light-induced absorption difference spectroscopy was performed to investigate the primary conformational changes of the purified proteins. The change in light-induced static absorption was measured using a Schinco spectrophotometer (Korea). Briefly, purified protein samples were kept in the dark for 15 min before the experiment, next, during 1 min of light illumination measurements were acquired every 800 ms for a full spectrum scan and 20 ms for a selected wavelength of interest. The same concentration of GR as HTH transcription factor protein and BSA was prepared in 50 mM Tris and 150 mM NaCl at pH 7.0. The average of each dataset was used for the fitting process. Based on the complex formation ratio by isothermal titration calorimetry, a GR-GvTcR complex was formed via 1:1 binding.

### Reporter system

For the luciferase reporter system, we inserted the promoter DNA sequence into multiple cloning sites (MCS) of pGL4.10[luc2] by cloning with the restriction enzymes *KpnI* and *XhoI*. A promoter cloned vector and a vector cloned using GR and GvTcR were co-transformed into the reporter *E. coli* (BL21 DE3 strain). This was inoculated and grown at 37 °C and 200 rpm overnight and then incubated until an OD of 0.5 was achieved through 1% transferred cells. Ampicillin, IPTG, and All-*trans* retinal (when GR was included) were added to a 24-well plate, mixed with cells, and induction was conducted for 2 h. Cells were assessed using a luminometer (Berthold) and the Luciferase Assay System kit (E1500; Promega, Madison, WI, USA). For the fluorescent reporter system, GFP was introduced using *NdeI* and *NotI* into the pET28a vector. We co-transformed the vector created by introducing promoter sequences at *BglII* and *XbaI*, and the vector expressing GvTcR or GR to the reporter *E.coli* (BL21 DE3 strain). This was inoculated and grown at 37 °C and 200 rpm overnight, and then grown until an OD of 0.5% was achieved through 1% transferred cells. Ampicillin, IPTG, and All-*trans* retinal (when GR was included) were added to a 24-well plate, mixed with cells, and induction was conducted for 2 h. Cell fluorescence was measured using a 2300 EnSpire Multimode Plate Reader (PerkinElmer, Waltham, MA, USA). The promoter sequence use is as follows: Each sequence was inserted by 5′-GGGCGCGCGGCGTCCAAT CAAGCAGAAGGCACTTACGGAAGCAACTCGCT-3′ for the promoter of the ABC transporter ATP-binding protein and 5′-CGGGGGACTTGACGGCGGCGCCCGGAAGTCATATAACTGTTTTTT TATATAAATTGCTGGTT-3′ for the GvTcR promoter.

### Real-time PCR

*Gloeobacter violaceus* PCC 7421 was obtained from the Culture Collection of Algae and was grown in a Z-medium under photoautotrophic conditions at 25 °C, 150 rpm. The light source was a fluorescent lamp (FL20SD) at an intensity of 10 µmol m^−2^ s^−1^. *G. violaceus* PCC 7421 was adapted to dark conditions for 3 days. For the light-to-dark experiment, samples were prepared by adapting cells to light conditions. A sensitivity test was performed using a prepared primer set. 16s rRNA was used as endogenous control. The primer sequence is as follows. Forward primer: 5′-CCTGACGGTACCTGACGAAT-3′, Reverse primer: 5′-GGTTGGCTAGAGTGCGGTAG-3′. Samples were obtained every hour, depending on the presence or absence of light, according to each condition, and cDNA synthesis was performed immediately for RT-PCR. One-Step PrimeScript™ III RT-qPCR Mix (Takara, Japan) was used to synthesize cDNA. RT-PCR was performed using an Applied Biosystems 7500 Real-time PCR system (Thermo Fisher Scientific, Waltham, MA, USA). The PCR reaction mixtures were prepared with 1.6 nM forward and reverse primers for each gene, 200 ng cDNA, and TOPreal™ SYBR Green qPCR 2X PreMIX (Enzynomics, Daejeon, Korea) in a total volume of 20 μL. The RT-PCR conditions were 95 °C for 10 min, followed by 30 cycles of 95 °C for 10 s, 53 °C for 1 min, and 97 °C for 2 min. The 16 s rRNA was used as endogenous control, and data was collected during the annealing step.

### Statistics and reproducibility

In proton pumping experiments were performed at least three times. Data are shown as boxplot ± s.d. (*n*   =   3). Absorption spectra data were fitted with multiple peak distribution (Gaussian) association curves, using Origin Pro 9.0. The fluorescent reporter system results were performed at *n* = 15 biologically independent samples. The luciferase reporter system results were performed at *n* = 8 biologically independent samples. Real-time PCR results were performed at *n* = 4 biologically independent samples.

### Supplementary information

Supplementary Information

Description of Additional Supplementary Files

Supplementary Data 1

reporting-summary.

## Data Availability

All data supporting the findings of this study are available within the paper and its Supplementary Information. Fluorescent reporter system for transcriptional regulatory promoter identification vector (Addgene ID : 221817, 221816). The source data behind the graphs in the paper can be found in Supplementary Data 1.
