# Supplementary material for: Proton-pumping photoreceptor controls expression of ABC transporter by regulating transcription factor through light
Source: Commun Biol. 2024 Jun 29;7:789. doi: 10.1038/s42003-024-06471-4 (PMC11217422; doi:10.1038/s42003-024-06471-4)
Supplement: Supplementary file 1 — Supplementary Information [file 42003_2024_6471_MOESM1_ESM.docx]

Supplementary Information

**Proton-pumping photoreceptor controls expression of ABC transporter by regulating transcription factor through light**

Jin-gon Shim ^1,2^, Kimleng Chuon ^1^, Ji‐Hyun Kim ^1^*,* Sang-ji Lee ^1^*,* Myung-chul Song ^1,3^, Shin-Gyu Cho ^1,3^*, Chenda Hour* ^1^, Kwang*-*Hwan Jung ^1,*^

^1^ Department of Life Science, Sogang University, Seoul, South Korea

^2^ Present address: Pharmacology Department, Northwestern University Feinberg School of Medicine, Chicago, Illinois USA

^3^ Research Institute for Basic Science, Sogang University, Seoul, Korea

*Corresponding Author

To whom correspondence should be addressed. Tel.: +82-02-705-8795. Fax: +82-02-704-3601. E-mail: [kjung@sogang.ac.kr](mailto:kjung@sogang.ac.kr)


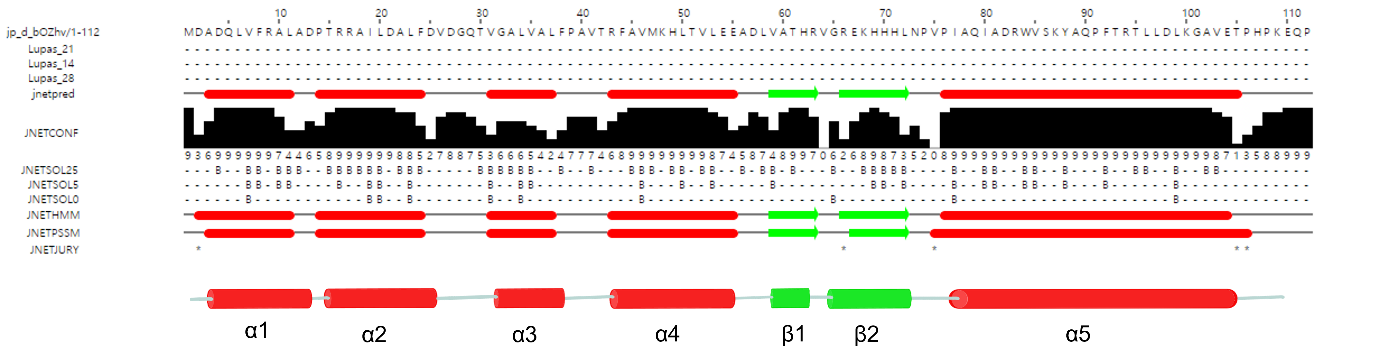


**Supplementary Figure S1** | **2D structure prediction of GvTcR.** The 2D secondary structure of GvTcR was predicted through JPred4, a protein secondary structure prediction server, and the results of the 5 alpha-helix and 2 beta-strand structures were shown. Alpha Helix is marked in red box, and green box is beta-strand.


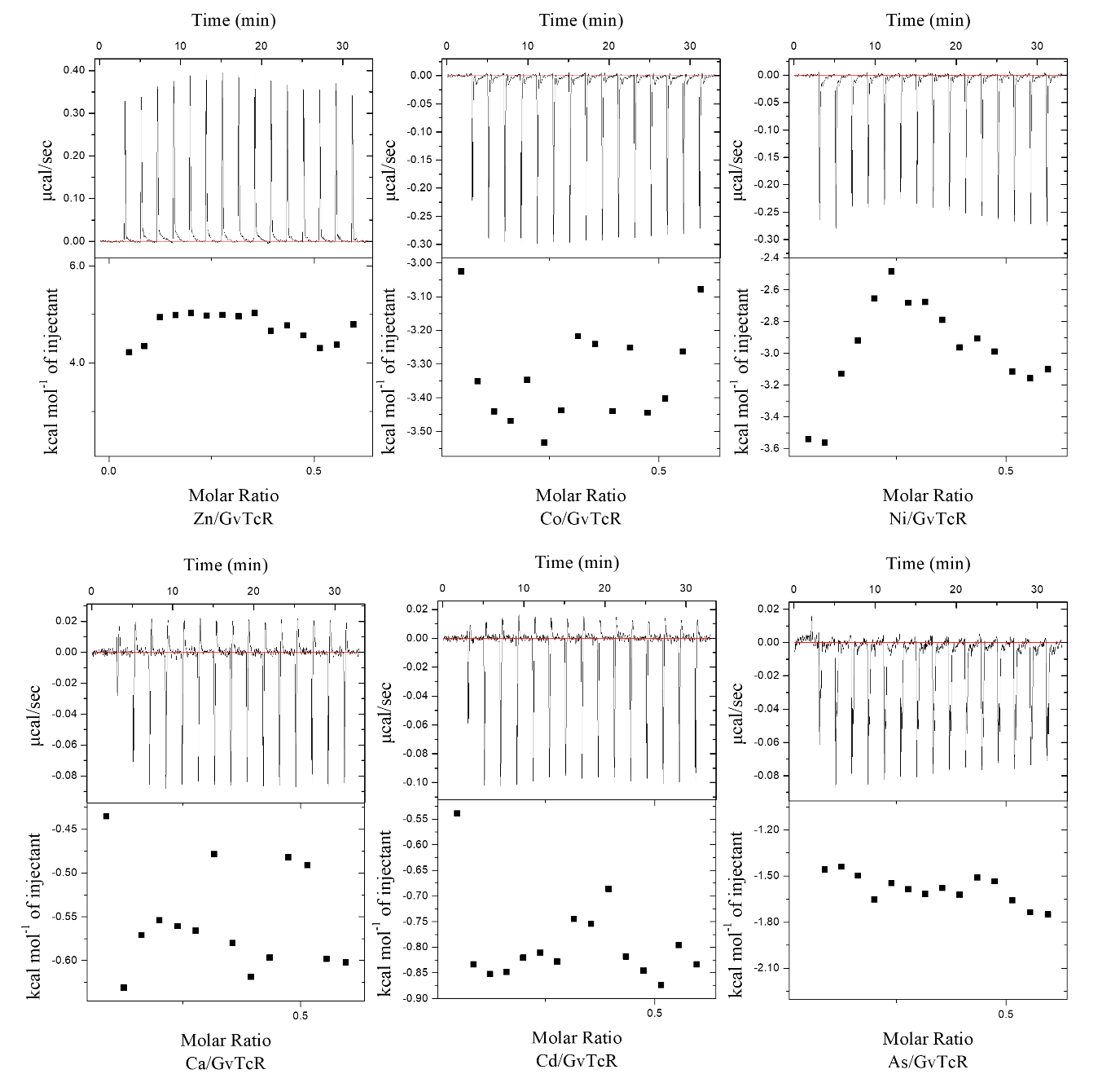
 **Supplementary Figure S2**| **Isothermal titration calorimetry (ITC) analysis for GvTcR with metal ions.** Isothermal titration calorimetry (ITC) analysis shows the results for GvTcR with various metal ion. A total of six were measured as Zn, Co, Ni, Ca, Cd, and As metal ions. The top and bottom panels show the raw data and the enthalpy changes. The fitting result is shown in the bottom panel as a continuous line. These experiments were carried out at room temperature.

**
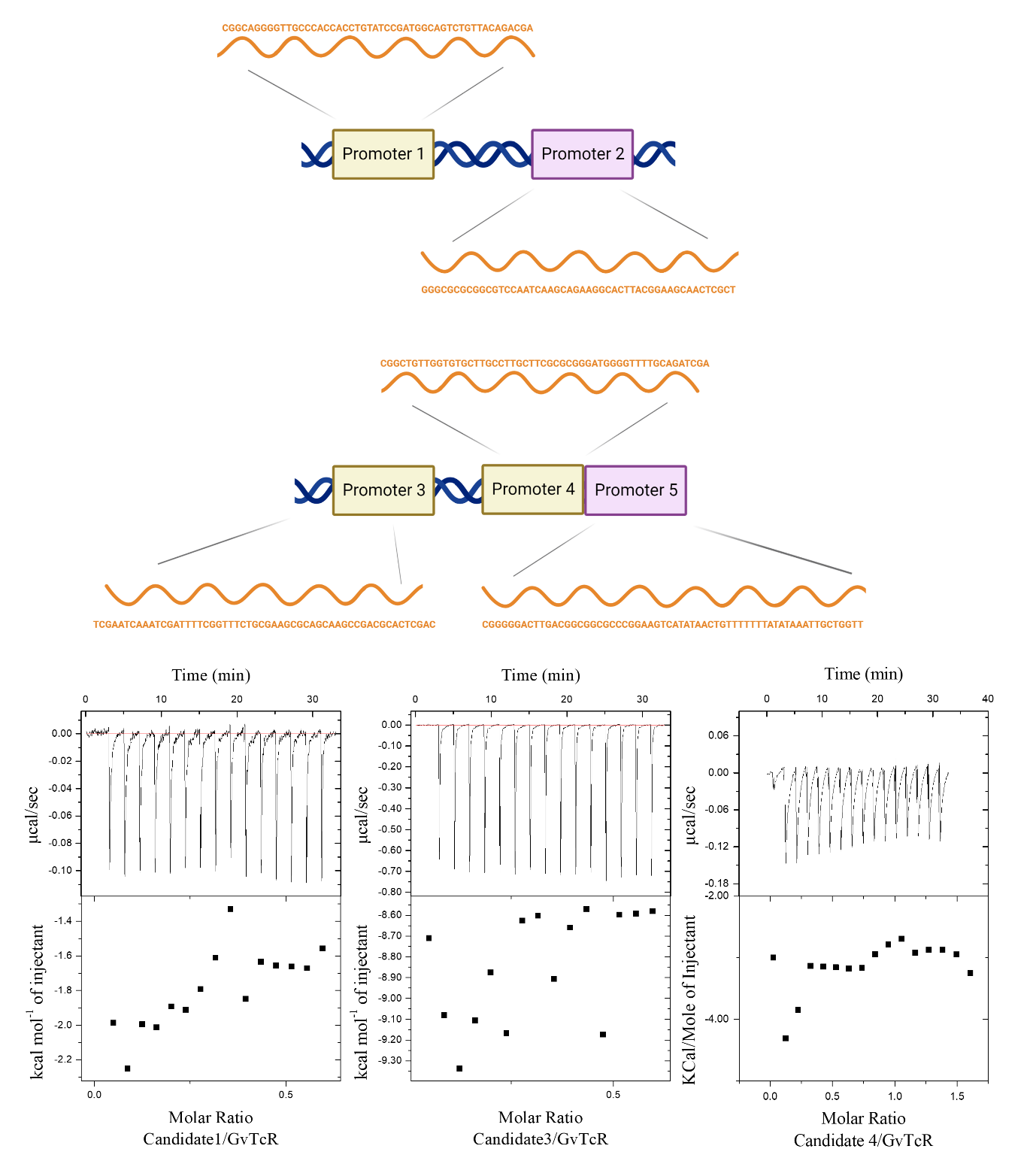
 Supplementary Figure S3**| **Information on predicted promoter regions and isothermal titration calorimetry (ITC) analysis for GvTcR with DNA fragments.** To determine the promoter regions of regulated gene that can binding with GvTcR, a total of five promoter region were predicted using Promoter Hunter, a promoter prediction server. Among these candidate groups, 1, 3, and 4 obtained unbound results. Candidate groups 1 and 2 are the groups in front of the ABC-transporter ATP binding protein gene and 3, 4, and 5 are the groups in front of GvTcR gene.


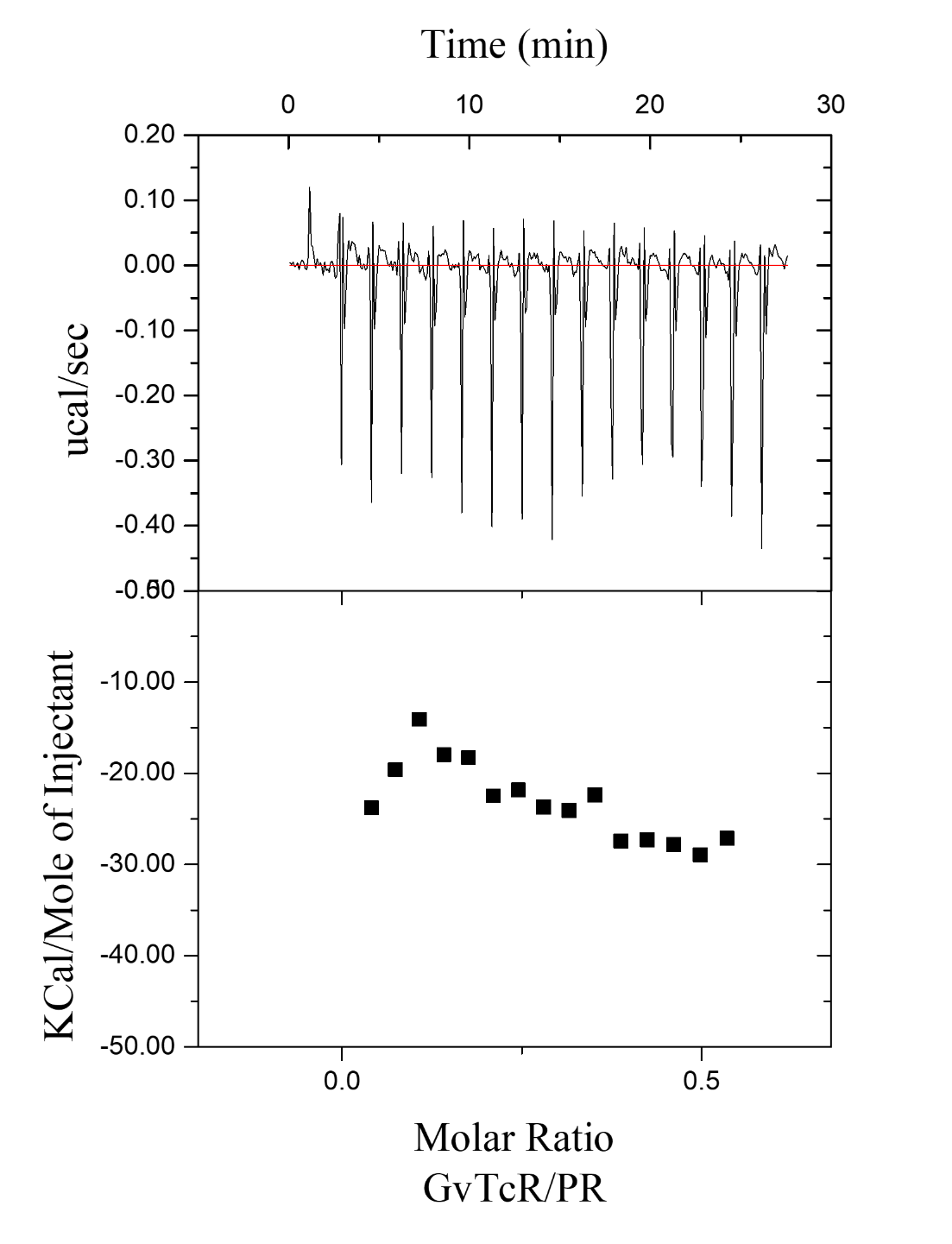


**Supplementary Figure S4**| **Isothermal titration calorimetry (ITC) analysis for GvTcR with PR.** Isothermal titration calorimetry (ITC) analysis shows the results for GvTcR with PR for control. The top and bottom panels show the raw data and the enthalpy changes. The fitting result is shown in the bottom panel as a continuous line. These experiments were carried out at room temperature.


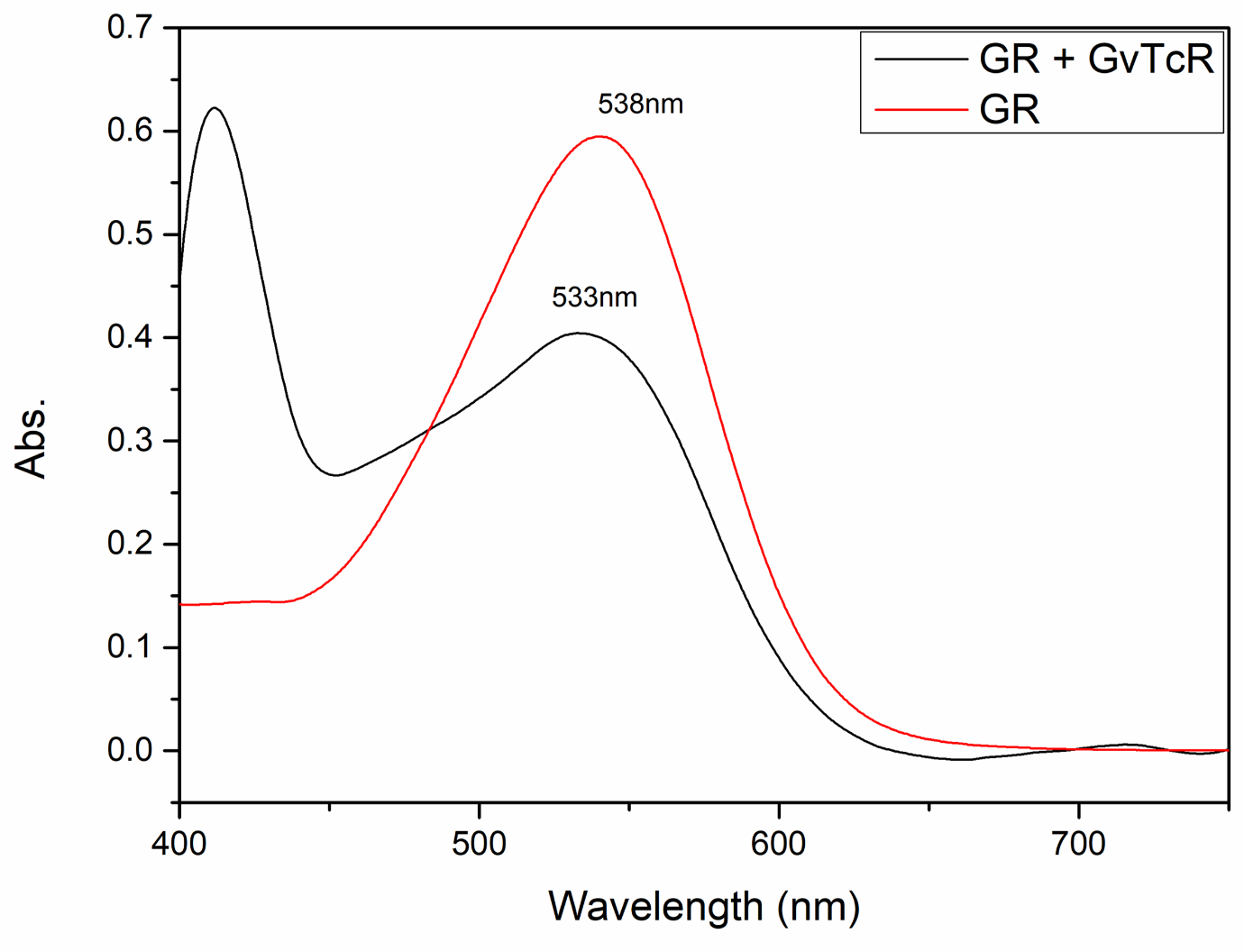
 **Supplementary Figure S5**| **Spectroscopic analysis of GR co-expressed with GvTcR.** A vector capable of more safely controlling polycistronic gene expression was prepared by introducing a ribosome binding site to one protein expression vector. The spectral shift was measured through the co-expression of GR and GvTcR. The spectral shift was measured at 533 nm through co-expression at 538 nm. The absorption spectrum of GR is indicated in the red line, and GR co-expressed with GvTcR is indicated in the black line.


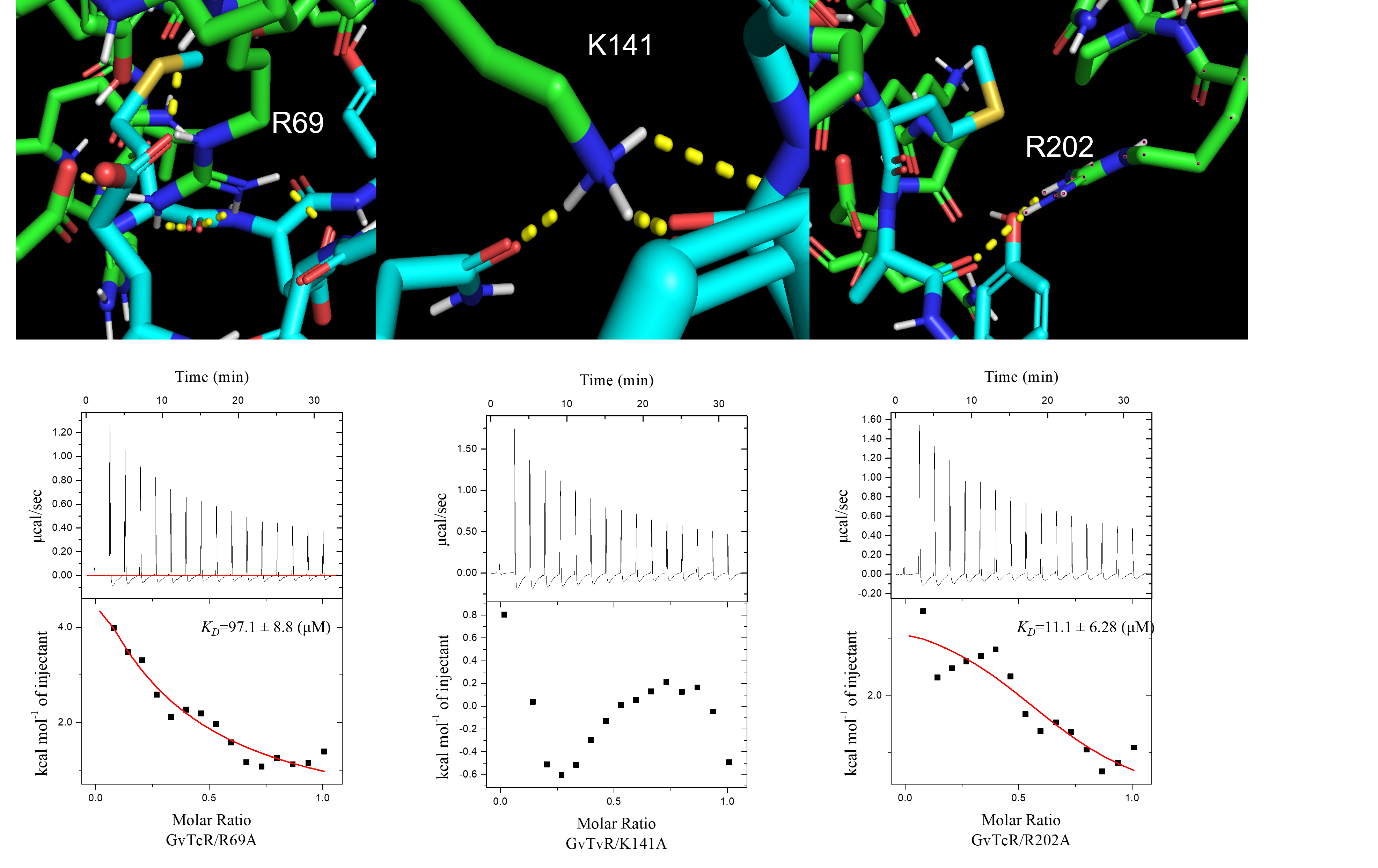


**Supplementary Figure S6**| **Binding site prediction through protein-protein docking simulation and isothermal titration calorimetry (ITC) analysis for mutants.** The combination of GR and GvTcR was predicted through Cluspro 2.0, a protein docking server. The positions of candidate groups R69, K141, and R202 capable of polar interaction were selected through PyMol (upper lane), and ITC results were obtained (lower lane). R69A mutant was measured as *K_D_* =97.1±8.8 μM, R202A mutant was measured as *K_D_*=11.1±6.28 μM, and K141A was not calculated. The top and bottom panels show the raw data and the enthalpy changes. The fitting result is shown in the bottom panel as a continuous line. These experiments were carried out at room temperature.


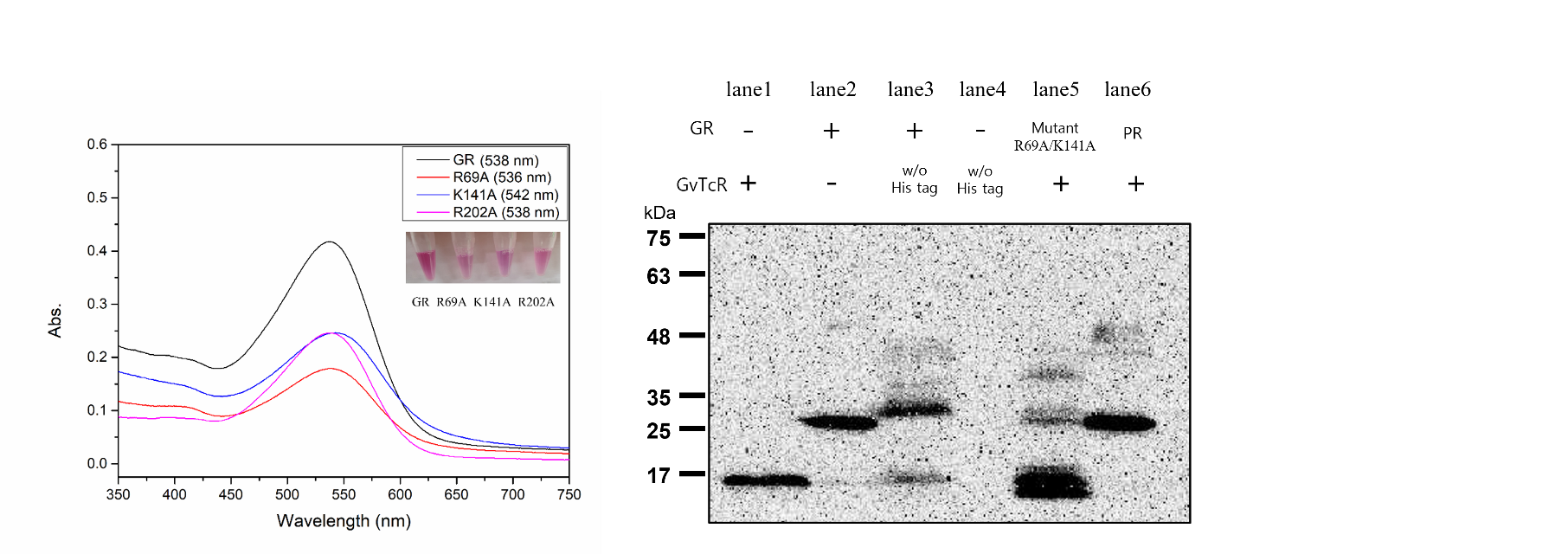
 **Supplementary Figure S7**| **The absorption spectra and protein pull-down assay for GR mutants.** The absorption spectrum for GR mutants was measured. GR is 538 nm and marked in black. The R69A mutant was measured at 536 nm and marked with a red line. K141A and R202A were measured at 542 nm and 538 nm, respectively, and marked with blue and pink. To measure protein-protein binding, pull-down assays were performed by Western blot assay. GvTcR-expressing cell lysate was blotted onto GR-bound beads, and then GR was extracted and subjected to Western blot. Lanes 1 and 2 are controls with bands of His-tagged GvTcR and GR. Lane 3 is the result of passing cell lysate expressing GvTcR without His tag, which showed a band higher than the band of GR alone. Lane 4 is the control of GvTcR without His tag. Lane 5 is the result of GR R69A/K141A mutant and His-tagged GvTcR, and lane 6 is the result of PR and His-tagged GvTcR.


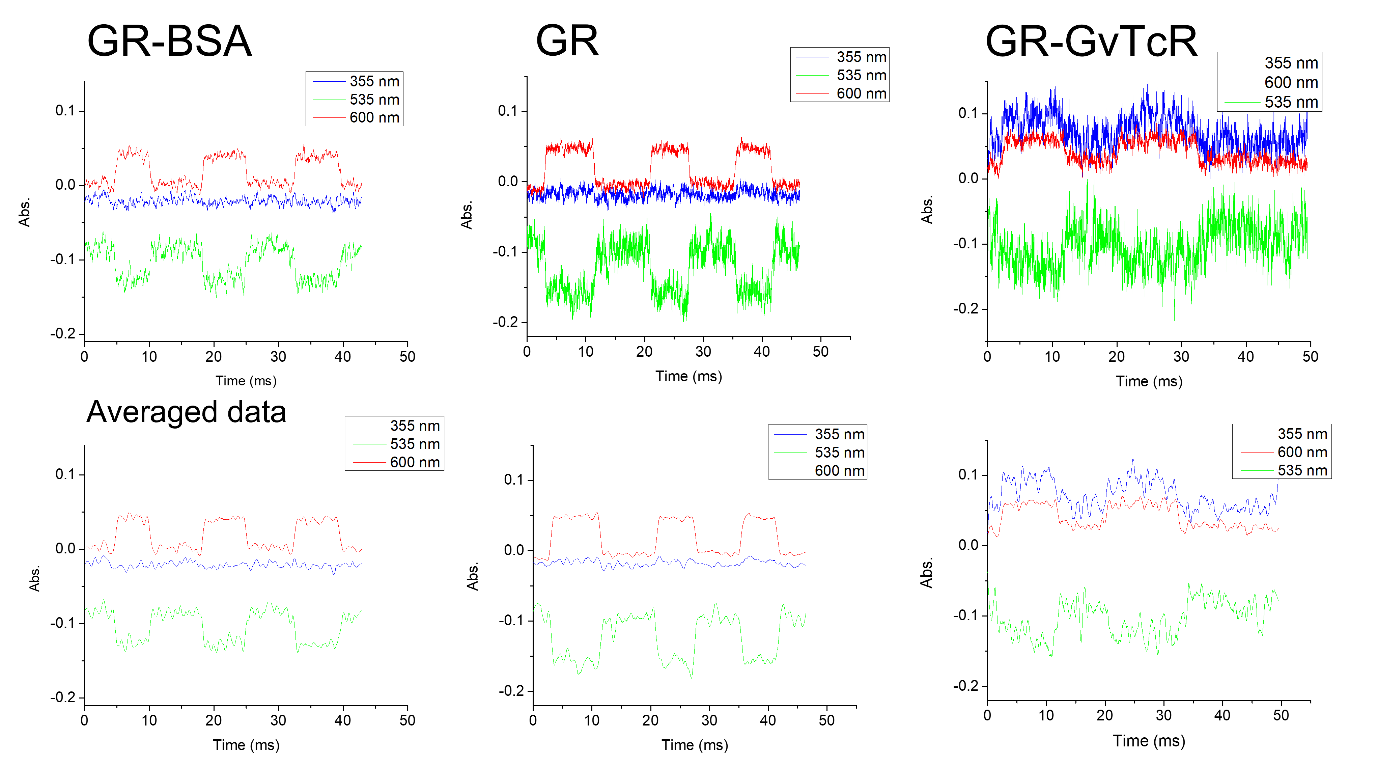
 **Supplementary Figure S8**| **Light dependent differential absorption spectrum of GR.** The light dependent differential absorption spectrum of GR mixed with BSA (left), only GR (middle), and GR bind to GvTcR (right). The continuous dark and light absorption change of the three sample groups were monitored at 355 (blue line), 535 (green line), and 600 nm (red line). Raw data are shown in the upper panel, and average data are shown in the lower panner. GR-BSA and GR-only samples showed fast excitation and relaxation upon light (white bar) and dark (black bar) in a similar manner. GR-GvTcR showed significantly higher absorption change at 355 nm along with slow recovery of ground absorption at 535 nm.


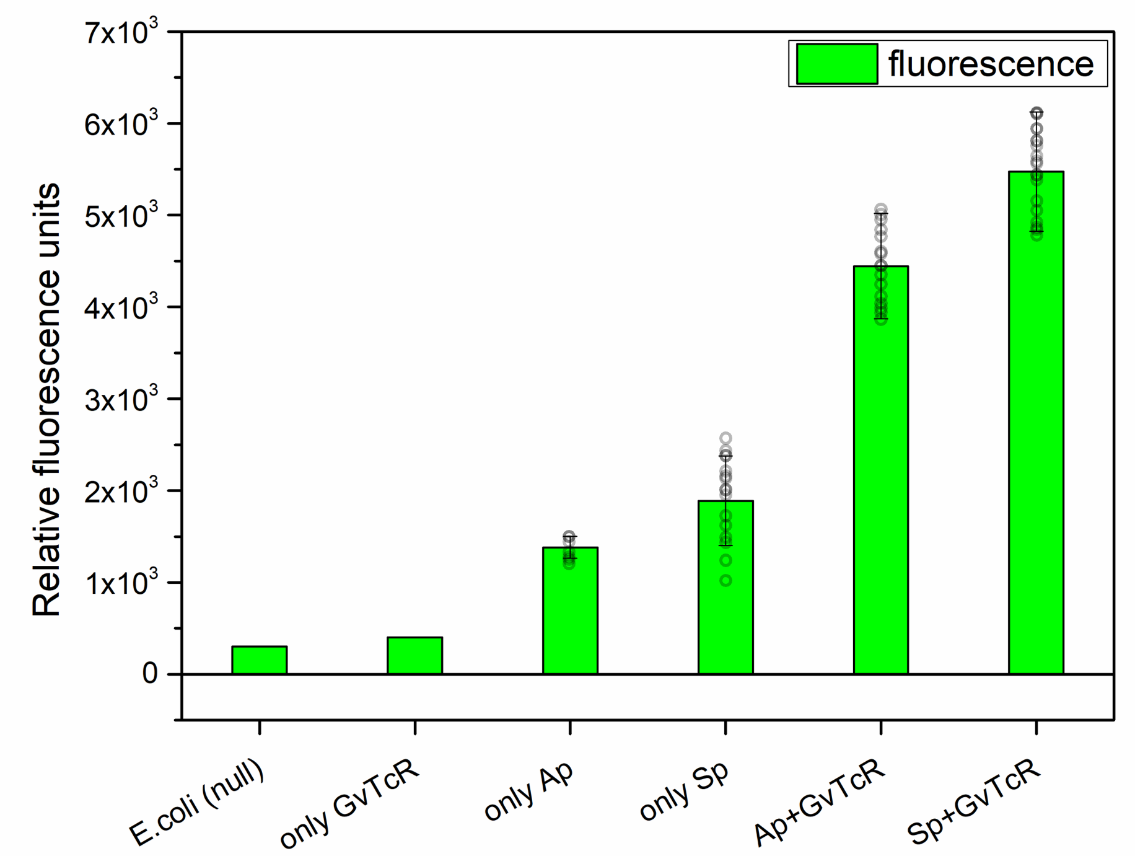


**Supplementary Figure S9**| **Functional measurement of GvTcR via a fluorescent reporter system.** Fluorescence levels were measured under various conditions through a fluorescence reporter system. The fluorescence of GFP, a reporter protein due to the function of GvTcR, was measured through fluorescence levels of *Escherichia coli*, Candidate promoters (1P, 3P) that do not bind GvTcR, only vector, GvTcR without promoter, and promoters with GvTcR. n=14 biologically independent samples.


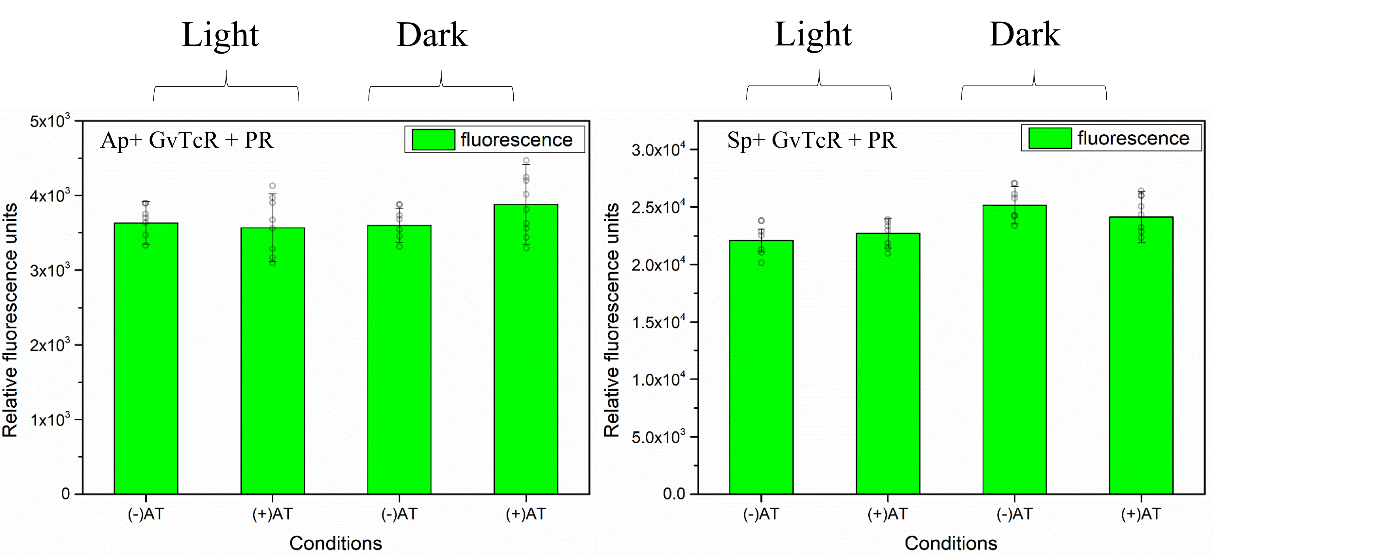
 **Supplementary Figure S10**| **Fluorescent reporter assays measured by coexpressions of PR and GvTcR.** The function of GvTcR according to the presence or absence of light was compared through PR. Along with the expression of PR, the regulation of GvTcR was measured through the presence or absence of a retinal chromophore. PR opsin without retinal and PR with retinal chromophore were compared in dark and light. Differences in fluorescence levels were compared. n=8 biologically independent samples.
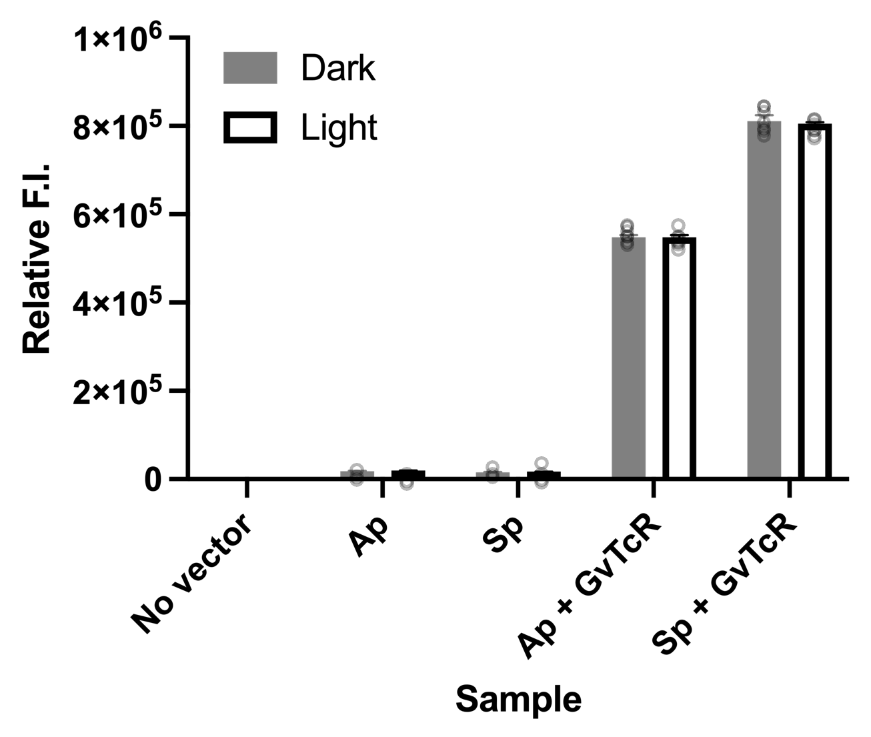


**Supplementary Figure S11**| **Functional measurement of GvTcR via a Luciferase reporter system.** Luminescence levels were measured under various conditions through a luciferase reporter system. The luciferase, a reporter protein due to the function of GvTcR, was measured through luminescence levels of *Escherichia coli*, no vector, only promoter, and promoters with GvTcR. n=13 biologically independent samples.


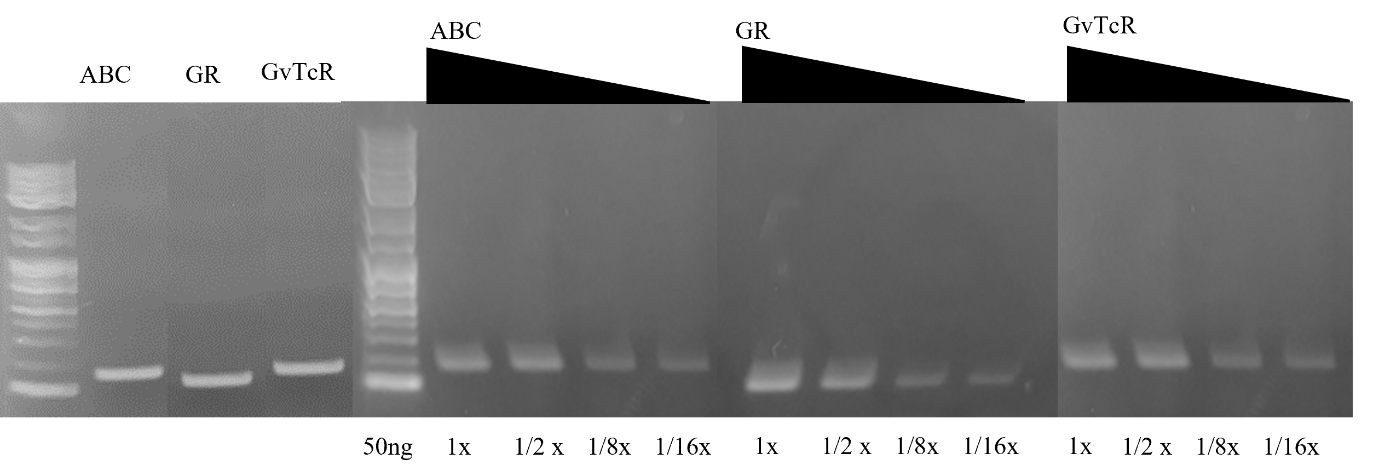


**Supplementary Figure S12**| **Sensitivity of primer set test for RT-PCR.** Detection test for the species-specific primers using template DNA from Gloeobacter Violacaeus. 50 ng was designated as 1x and indicated in the lane for each dilution.
